# The cost of caring: Gendered health and labor market effects of grandparenthood

**DOI:** 10.1073/pnas.2535409123

**Published:** 2026-05-29

**Authors:** Maria Lyster Andersen, Rannveig Kaldager Hart, Hans Fredrik Sunde, Neil Martin Davies, Fartein Ask Torvik

**Affiliations:** ^a^ Centre for Fertility and Health, Norwegian Institute of Public Health, Oslo 0476, Norway; ^b^ Department of Psychology, University of Oslo, Oslo 0373, Norway; ^c^ Department of Health Management and Health Economics, Institute of Health and Society, University of Oslo, Oslo 0373, Norway; ^d^ Division of Psychiatry, Department of Statistics, University College London, London W1T 7NF, United Kingdom; ^e^ Department of Public Health and Nursing, Norwegian University of Science and Technology, Trondheim 7491, Norway; ^f^ Promenta Research Center, Department of Psychology, University of Oslo, Oslo 0373, Norway

**Keywords:** grandparenthood, retirement, fertility, health, gender

## Abstract

While the effects of the transition to parenthood are well researched, less is known about how the transition to grandparenthood affects health and labor market outcomes. Using comprehensive Norwegian register data covering the entire population born between 1950 and 1960, we examine the effects of first-born grandchildren born during 2007–2018. Employing event-study models with person-year records, we compare grandparents to not-yet grandparents. Our findings reveal a sharp increase in the likelihood of respiratory infections during the first two years of grandparenthood, with infections increasing by 56% for women and 31% for men. Additionally, grandparenthood modestly reduces the likelihood of doctor’s visits related to mental disorders (4.5%) and cardiovascular health (3.3%). Grandmothers also see a decline in musculoskeletal-related visits (3.8%). These health-related changes coincide with notable gendered effects on labor market participation. Ten years after the birth of their first grandchild, employed women are 12% less likely to hold full-time positions compared to a 2% reduction for men. Overall, our findings demonstrate that the transition to grandparenthood significantly reshapes health and economic outcomes for both women and men. The larger effects observed for women likely reflect their greater involvement in informal childcare provision. Our results underscore the intersection of health, family dynamics, and gendered labor market behaviors in late adulthood.

## Significance

Becoming a grandparent is a major life transition, yet its consequences for health and work are not fully understood. Using population-wide Norwegian register data, we document how becoming a grandparent alters both health service use and labor market attachment. We show that grandparenthood increases respiratory infections, while slightly reducing consultations for mental, cardiovascular, and musculoskeletal conditions. At the same time, it leads to substantial and persistent reductions in full-time employment and earnings, especially for women. These findings highlight how family expansion in late adulthood shapes health and gendered employment trajectories.

Becoming a parent and a grandparent are important life events, but many countries now see a growing share of childless adults, and those who become parents have their first child later in adulthood. These changes will also result in changes to grandparenthood. More childless adults imply that a greater share of parents will never become grandparents (1). As many older adults value grandparenthood (2), this could reduce protective health factors. Parents generally have better health than childless individuals (3–5), but it is unclear whether this advantage extends to grandparents. At the same time, the relationship between women’s fertility and labor force participation has also changed (6–8). More women can now combine having a family and working (9, 10). This trend is not limited to mothers, but extends to grandmothers, who today are more likely to be employed. As their offspring often combine work and childrearing, grandparents’ time could be a sought-after resource, with potential labor market consequences. The importance of parenthood has been extensively studied, but less is known about how grandparenthood relates to health and labor market outcomes. Given the large changes in fertility behavior, labor market institutions, and their implications for grandparenthood, we need to better understand how grandparenthood impacts important aspects of life, including health and labor market outcomes.

A study of ten Western European countries found that grandparenthood on average led to reductions in well-being, but not in physical, cognitive, and mental health (11). However, this average effect masks some heterogeneity. Grandparents who had less family contact and were less involved in childcare typically saw reductions in well-being, while those who were more involved saw some improved health and no reduced well-being. Similarly, a systematic review found that custodial grandparents (i.e. those with formal caregiving responsibilities) tended to fare worse. By contrast, non-co-residing grandparents often experienced positive associations between grandparenting, health, and well-being (12). A Norwegian registry study on grandparenthood and mortality also found significant differences within the group of grandparents. Mortality was higher among grandfathers and younger grandmothers, while women who entered grandparenthood later in life experienced slightly lower mortality relative to women without grandchildren (13). Taken together, these studies suggest that the health effectsof grandparenthood may depend on factors such as the timing of grandparenthood and the degree of contact with grandchildren.

In the long run, women bear the primary labor market costs associated with childbearing and childrearing (14, 15), whereas in many settings men experience a wage premium from parenthood (16). Because the first grandchild is often born before retirement, grandparenthood may likewise affect labor supply. In today’s cohorts of grandparents, men typically have higher earnings, while women on average have substantially more childcare experience. Becker’s theory of household specialization (17) therefore suggests that the transition to grandparenthood may induce a gendered reallocation of paid and unpaid work, with grandmothers more likely than grandfathers to reduce market work in order to provide care. This prediction is further reinforced by theories of gendered roles and expectations, as many women continue to take greater responsibility for housework and care even when they out-earn their partner (18, 19). Recent evidence on labor market effects of grandparenthood from Denmark and Sweden points in different directions. In Denmark, grandmothers’ labor market participation declined more than grandfathers’, widening the gender wage gap (20, 21), whereas in Sweden grandparenthood had no clearly gendered effect on retirement (22). Given the institutional similarities between these countries, these divergent findings suggest that the labor market consequences of grandparenthood may depend on context and cohort-specific characteristics. Nonetheless, the results from these studies underscore how grandparenthood may contribute to a second gender wage gap if retirement effects are gendered. In many settings, including in Norway, changing the timing of one’s retirement by a few years can have large repercussions for later pension payments. Consequently, a small, gendered effect of grandparenthood on the timing of retirement could translate to much greater gender gaps in future income and wealth.

Grandparenthood may also affect health, both through psychosocial mechanisms and through changes in labor supply. Becoming a grandparent may alter social roles, daily routines, and perceived meaning in ways that improve well-being. Grandparenthood can increase feelings of meaning and make aging more salient (23–25), which may strengthen motivation for health-promoting behavior. In contrast to parenthood, which has been shown to worsen mental health at least in the short run (26) and to reduce time available for health-promoting activities (27), grandparental care can be less intensive, more voluntary, and more easily adjusted to capacity. Grandparenthood may therefore provide emotional rewards and opportunities for meaningful engagement without the same obligations that accompany parenthood. Furthermore, grandparenthood may also affect health through changes in labor supply. If becoming a grandparent leads some individuals to reduce working hours or retire, health may improve through reduced work-related stress and increased time for health-promoting behaviors (28–31).

In sum, we expect that grandparenthood may improve both mental health and somatic health conditions plausibly related to stress and lifestyle, such as musculoskeletal and cardiovascular disorders. At the same time, more frequent contact with young children may increase exposure to communicable diseases. Effects of grandparenthood on health and labor market outcomes are further likely to depend on caregiving intensity and prior labor market attachment. If grandmothers provide more care than grandfathers and are more likely to reduce work hours or retire, the effects of grandparenthood on both health and labor supply should be stronger for women. In this case, grandparenthood could amplify the gender health gap in favor of women (32, 33) while also widening the gender earnings gap in favor of men (34).

Access to Norwegian registry data gives us a unique opportunity to study how grandparenthood relates to health and labor market outcomes across entire birth cohorts, without participation bias. We focus on individuals born between 1950 and 1960 and examine two related questions: first, descriptive differences between individuals with and without grandchildren, and second, the causal effects of grandparenthood using a staggered event-study design. Although parental characteristics affect the likelihood of having children, they are more strongly related to parenthood than to the exact timing of grandparenthood. Therefore, one can consider the exact timing of grandparenthood as more random, with some notable exceptions. For the event-study, we will look at health and labor market outcomes in the period leading up to and after the first grandchild’s birth. As this approach means comparing people with themselves, we will avoid issues related to the selection into grandparenthood and time-invariant confounding. Based on prior work and the mechanisms outlined above, we focus on health outcomes that are plausibly responsive to changes in lifestyle, stress, and social role following the transition to grandparenthood. We also examine a condition less likely to respond to such changes in the short run as a placebo outcome (endocrine and metabolic health). Finally, the richness of the registry data allows us to look at how factors such as gender, socioeconomic status, and geographical proximity between grandparents and grandchildren shape these associations.

We investigate how grandparenthood is related to health and labor market outcomes by addressing the following research questions:

**1**: How does grandparenthood affect selected physical and mental health conditions?
**2**: How does grandparenthood affect income and labor market participation?
**3**: Are health and labor market effects independent of each other?
**4**: Do gender, socioeconomic status, and geographical distance moderate any of these relationships?

## Results

### Associations Between Grandparenthood, Health, and Labor Market Outcomes.

Table 1 shows how the health and labor market characteristics of grandparents compare to the characteristics of those without grandchildren (excluding childless individuals). First, we see that grandparents are slightly older. This is likely a result of there being a higher share of the oldest birth cohorts in the grandparent group. In terms of primary health care use, there is a very small difference between the two groups. Grandmothers are slightly less likely to have health care visits due to mental health complaints or mental disorders. This difference in mental health-related visits is smaller among men. At the same time, both grandparents are slightly more likely to see a doctor in relation to cardiovascular health.

**Table 1.** Descriptive Statistics: Comparing men and women with and without grandchildren (Norwegians born in 1950–1960, grandchildren born 2007–2018)

	0 Grandchildren	1+ Grandchildren	Diff.	SE
Women				
Age	56.11	56.73	0.62	(0.007)
Married	0.54	0.67	0.13	(0.0007)
Income (CPI adjusted, NOK)	366,884	352,643	−14,241	(451)
Works full-time	0.61	0.56	−0.05	(0.0009)
PHC visit related to CVD	0.18	0.20	0.02	(0.0006)
PHC visit related to MH	0.20	0.18	−0.02	(0.0005)
PHC visit related to EH	0.17	0.17	0.002	(0.0005)
PHC visit related to MSH	0.45	0.48	0.03	(0.0007)
Mental disorders	0.09	0.08	−0.01	(0.0004)
Symptoms of mental illness	0.13	0.13	−0.007	(0.0005)
Cardiovascular disease	0.15	0.17	0.02	(0.0005)
Cardiovascular symptoms	0.03	0.04	0.01	(0.0003)
Endocrine disease	0.16	0.16	0.002	(0.0005)
Endocrine symptoms	0.008	0.008	0	(0.0001)
Respiratory infections	0.15	0.18	0.03	(0.0005)
Education				
- Compulsory	0.19	0.19	0.0006	(0.0005)
- High School	0.40	0.45	0.05	(0.0007)
- University	0.41	0.36	−0.05	(0.0007)
Men				
Age	56.18	57.19	1.01	(0.006)
Married	0.58	0.68	0.10	(0.0006)
Income (CPI adjusted, NOK)	600,170	581,569	−18,601	(853)
Works full-time	0.90	0.89	0.004	(0.0005)
PHC visit related to CVD	0.22	0.25	0.03	(0.0005)
PHC visit related to MH	0.11	0.11	−0.01	(0.0004)
PHC visit related to EH	0.13	0.14	0.01	(0.0004)
PHC visit related to MSH	0.36	0.38	0.02	(0.0006)
Mental disorders	0.05	0.05	−0.005	(0.0003)
Symptoms of mental illness	0.08	0.08	0.005	(0.0004)
Cardiovascular disease	0.20	0.23	0.03	(0.0005)
Cardiovascular symptoms	0.03	0.03	0.003	(0.0002)
Endocrine disease	0.13	0.14	0.01	(0.0004)
Endocrine symptoms	0.005	0.005	0	(0.00009)
Respiratory infections	0.10	0.11	0.01	(0.0004)
Education				
- Compulsory	0.17	0.18	0.01	(0.0005)
- High School	0.46	0.52	0.06	(0.0005)
- University	0.37	0.30	−0.07	(0.0006)
Observations	1,690,668	2,983,400		

Calculated for the period 2006–2019. Childless individuals are excluded from the comparison. PHC: primary health care, CVD: cardiovascular disease, MH: mental health, EH: endocrine health, and MSH: musculoskeletal health. 2015 is used as the base year for inflation adjustment of income.

On the other hand, there appears to be bigger differences in terms of labor market outcomes. Among both men and women, grandparenthood is associated with lower annual incomes. The income difference is largest, both in absolute and relative terms, for women. Grandmothers have on average around NOK 14,240 lower annual incomes compared to those without grandchildren, which translates to a ≈4% decrease. Among the grandmothers, there also is a significantly lower share employed in full-time positions. Grandfathers are also slightly less likely to be in full-time employment. However, the difference is again greatest both in relative and absolute terms among women. Last, while the share with only compulsory education is similar in all groups, grandparents are less likely to have completed university education and more likely to have upper secondary as their highest level of educational attainment.

### Grandparenthood’s Effect on Health.

**Fig. 1.**
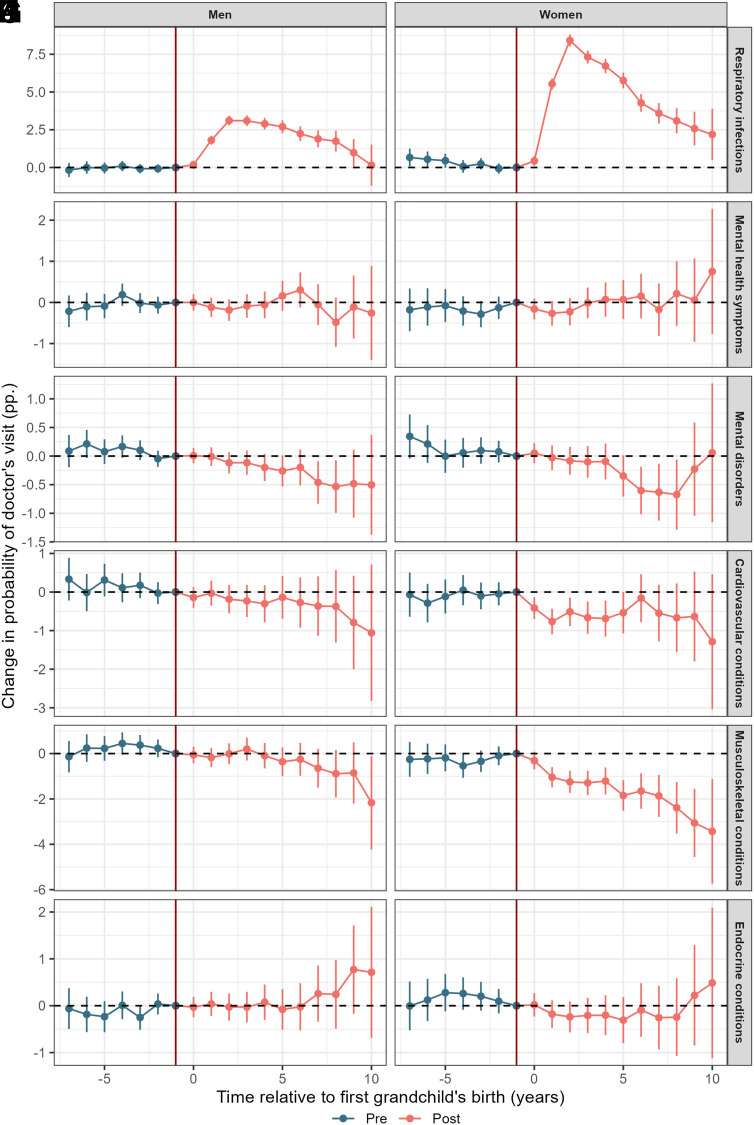
Average treatment effect of the birth of one’s first grandchild (at time 0) on respiratory infections (*A* and *B*), mental health symptoms (*C* and *D*), mental disorders (*E* and *F*), cardiovascular health conditions (*G* and *H*), musculoskeletal conditions (*I* and *J*), and endocrine conditions (*K* and *L*). The year prior to birth (−1) is used as the reference year. Individuals are born in 1950–1960, grandchildren born 2007–2018. Beside individual-specific effects, we control for year and birth year, and those not-yet treated (i.e. not-yet grandparents) constitute the control group.

Fig. 1 displays estimated changes in grandparents’ health from seven years prior to ten years following the birth of the first grandchild, separately for grandfathers and grandmothers. *SI Appendix*, Fig. S2 reports the corresponding estimates for the pooled sample without gender stratification.

#### Respiratory infections.

Panels (*A* and *B*) show how grandparents become significantly more likely to seek medical care due to respiratory infections in the year following the grandchild’s birth. For both grandparents, the highest likelihood of infections appears around two years after the first grandchild was born. The baseline likelihood of seeking health care related to respiratory infections in the year prior to grandparenthood was 14.9% for women and 10% for men. At the peak (t = 2), grandmothers were 8.4 percentage points, or 56% more likely to see a doctor due to respiratory infections. Grandfathers were 3.1 percentage points, or 31% more likely to see a doctor due to respiratory infections. While women were overall more likely to see a doctor due to respiratory infections, we still see a significant gender difference in the increase in doctor’s visits. We also studied specific respiratory infections such as acute upper respiratory infections and influenza, with similar results presented in *SI Appendix*, Figs. S3 and S4. *SI Appendix*, Fig. S5 shows similar results when we estimate the effect of grandparenthood on respiratory infections through the alternative IV-event design.

#### Mental health.

Panels (*C*–*F*) of Fig. 1 show how the likelihood of primary health care visits related to mental health changes in the period after the birth of one’s first grandchild. In the period leading up to birth, there appears to be no statistically significant differences in the mental health of those who will become grand- parents next and those who will become grandparents at a later time. With regard to symptoms (*C* and *D*), we also see no significant change in the mental health of grandparents compared to not-yet grandparents. All estimates are both insignificant and close to zero in magnitude. Turning to mental disorders (*E* and *F*), there appears to be some changes in the likelihood of mental disorders after entering grandparenthood. For all grandparents, we see a clear downward trend in the estimates after the first grandchild is born. In the early period of grandparenthood these effects are not significant. However, toward the end of our observation period the estimated effects are both negative and significant. 7 to 8 y after the transition to grandparenthood, grandmothers and grandfathers are about 0.5 to 0.6 percentage points less likely to see their GP due to mental disorders. While similar in absolute terms, given the different baseline probabilities these changes correspond to relative effect sizes of 7.2% (grandmothers) and 10% (grandfathers). As our estimates for mental disorders are consistently negative but with some uncertainty, we also consider the estimates from the pooled model with both grandparents included (*SI Appendix*, Fig. S2). With increased power, we now can detect significant effects slightly earlier. Overall, when comparing pre- and posttreatment effects, we find a small but significant effect, as grandparenthood decreases the likelihood of mental disorder-related visits by 0.3 percentage points. Given the baseline probability of 6.6%, this gives an effect size of 4.5%. *SI Appendix*, Fig. S6 shows the same model estimated specifically for depressive disorder where we also find similar results.

#### Cardiovascular health.

Panels (*G* and *H*) of Fig. 1 show how the likelihood of doctor’s visits related to cardiovascular health conditions changes with grandparenthood. In the posttreatment period, the point estimates are consistently negative, indicating an overall lower probability of cardiovascular-related doctor’s visits for both grandmothers and grandfathers. The estimates are modest in magnitude and somewhat uncertain for men, but we find a clearer decline in doctor’s visits among women. During the first years of grandparenthood, grandmothers are about 0.8 percentage points less likely to see a doctor due to cardiovascular issues. Given that the probability of such visits was 18.9% at baseline (t = −1), this translates to a relative effect size of 4.2%. For men, our estimates are more uncertain and also smaller in magnitude (−0.2 pp., or less than a 1% change). While Fig. 1 shows the results for all doctor’s visits stratified by genders, *SI Appendix*, Figs. S2, S7, and S8 show the results for both genders pooled together, as well as the results for the diagnoses and symptom-codes separately. We find no clear evidence that the results differ significantly by the code-grouping. However, we find that when the analysis is done without first stratifying by grandparent gender, we have more power to detect significant effects. In our pooled model, all estimates are statistically significant. Ten years after the transition, grandparenthood is estimated to reduce doctor’s visits by 1.6 pp., relative to a baseline probability of 21.2% (an effect size of 7.6%). Across all observed posttreatment years, grandparenthood reduced the likelihood of a doctor’s visit by an average of 0.7 pp. (3.3%).

#### Musculoskeletal health.

Panels (*I* and *J*) of Fig. 1 show the estimated effect of grandparenthood on musculoskeletal health. Grandmothers are significantly less likely to see their doctor with issues related to musculoskeletal health after their first grandchild is born. Ten years after their first grandchild, grandmothers are 3.4 percentage points less likely to have musculoskeletal health issues (effect size ≈7%, given the baseline of 47.9%). Over the entire observation period, the average effect is a 1.8pp reduction, or 3.8%. Overall, we find no significant effect for grandfathers.

#### Endocrine health.

Panels (*K* and *L*) of Fig. 1 show the estimated effect of grandparenthood on endocrine/metabolic health. In contrast to the previous health outcomes studied, we find no evidence of changing trends in the outcome after the first grandchild is born. This suggests that grandparenthood leads to neither fewer nor more frequent doctors visits due to causes like diabetes or weight change. In *SI Appendix*, Fig. S2 shows results from our pooled model, and *SI Appendix*, Figs. S9 and S10 show the results split by code-number (symptoms vs. disorders). We find no significant effect of grandparenthood in any of these models.

### Grandparenthood’s Effect on Labor Market Outcomes.

**Fig. 2.**
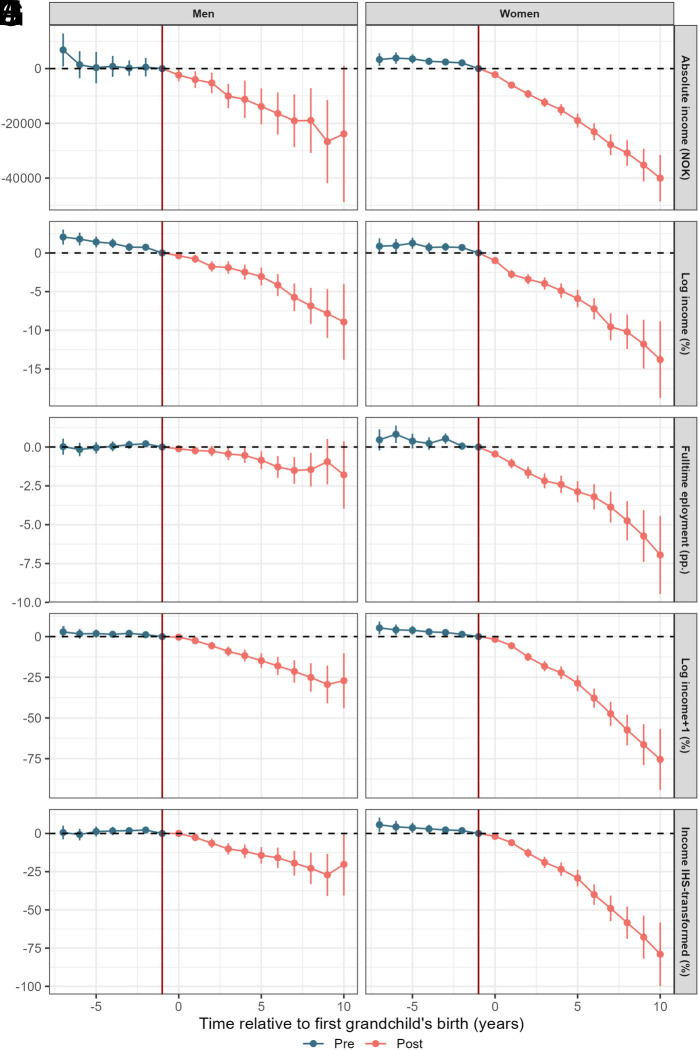
Average treatment effect of the birth of one’s first grandchild (at time 0) income in NOK (*A* and *B*), log-transformed income (*C* and *D*), full-time employment status (*E* and *F*), log transformed income+1 (*G* and *H*), and inverse hyperbolic sine (IHS) transformed income (*I* and *J*). The year prior to birth is used as the reference year. Individuals are born in 1950–1960, grandchildren born 2007–2018. Beside individual-specific effects, we control for year and birth year, and those not-yet treated (i.e. not-yet grandparents) constitute the control group.

Fig. 2 shows how grandparenthood impacts a variety of measures related to income and employment status.

#### Income.

Panels (*A*–*D*) of Fig. 2 show changes in absolute and relative incomes before and after the birth of the first grandchild. There appears to be significant income effects, and more so for women. Starting with absolute terms, panels (*A* and *B*) show how grandparenthood suppresses earnings. The average effect amounts to a 13,789 NOK decrease in earnings for men, and a 20,081 NOK decrease for women. However, the slope of the income change is much steeper for women. Consequently, ten years into grandparenthood, the decrease in earnings is now 40,038 NOK for grandmothers compared to not-yet grandmothers.

Looking at changes in relative terms (*C* and *D*), seven to ten years after becoming grandmothers, women are estimated to earn between 10 and 14% less than women who have not yet become grandmothers. In comparison, grandfathers earn about 6 to 9% less than not-yet grandfathers. As with changes in absolute income, grandmothers also see a steeper change in relative income than grandfathers. The greater impact on the grandmothers’ wages is also reflected in the widening intracouple wage gap (*SI Appendix*, Fig. S11). Because log-transformations exclude observations with zero income, the estimated effect of grandparenthood on logged income only captures intensive margin responses, i.e. income changes among individuals with positive earnings. When zeros are included in alternative specifications (*G*–*J*), the estimated effects become consistently larger, reflecting additional effects along the extensive margin (i.e. grandparents exiting the labor market).

#### Full-time employment.

While lower incomes likely reflect a lower degree of labor market participation, this may not always be the case. Consequently, panels (*E* and *F*) of Fig. 2 illustrate how the likelihood of being employed in a full-time position changes around the transition to grandparenthood—for the sample of employed individuals. As with income effects, we find some differences between the genders. While both men and women become more likely to transition to a part-time role after becoming grandparents, the magnitude of this effect is much stronger among grandmothers. After seven years of grandparenthood, the likelihood of holding a full-time position declines by approximately 1.5 percentage points for grandfathers and by 5 percentage points for grandmothers. This translates to an effect size of less than 2% for men but roughly 9% for women, given the average full-time employment rates in the year prior to grandparenthood (90.4% and 56.8%, respectively). Furthermore, while we see a continuing decline in the likelihood of full-time work for grandmothers throughout our observation period, the long-term effects are more uncertain among grandfathers.

### Are Health and Labor Market Effects Independent?

So far we have shown that grandparenthood has the potential to increase respiratory infections, reduce mental, musculoskeletal, and cardiovascular health issues, and lower income and employment levels. While it is difficult to imagine how increased infections could be caused by lower incomes or more part-time employment, it is possible that the other health effects of grandparenthood at least partly could be a by-product of less labor market participation and work-related stress.

**Fig. 3.**
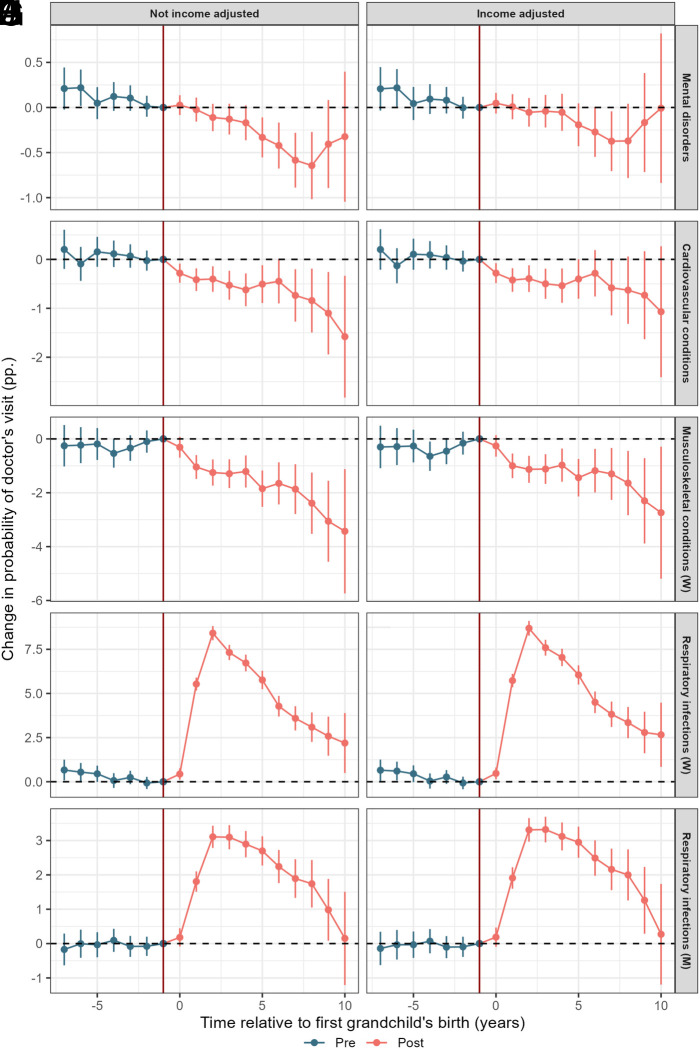
Average treatment effect of the birth of one’s first grandchild (at time 0) on mental disorders (*A* and *B*), cardiovascular conditions (*C* and *D*), musculoskeletal conditions in women (*E* and *F*), and respiratory infections (*G*–*J*). The year prior to birth is used as the reference year. Individuals are born in 1950–1960, grandchildren born 2007–2018. Beside individual-specific effects, we control for year and birth year, and those not-yet treated (i.e. not-yet grandparents) constitute the control group.

Panels (*A*–*D*) of Fig. 3 show how the estimated effects of grandparenthood on mental and cardiovascular health attenuate when we keep income fixed in our models. We consider income as a proxy for labor market participation. For mental health, when we compare the results presented in panels (*A* and *B*), we find near full attenuation, as the estimates become insignificant and move closer to zero. We also see some attenuation with regard to cardiovascular health. However, when comparing the results for cardiovascular health shown in (*C* and *D*), there still remains a clear downward trend in the posttreatment period even when adjusting for income. Looking at musculoskeletal health (*E* and *F*), we find very little attenuation. Similarly, when we adjust for income we find no evidence of attenuation of the effect of grandparenthood on respiratory infections (*G*–*J*). In fact, when controlling for income we see slightly larger estimated effect sizes for women. In the unadjusted model (*G*), grandmothers have 8.4 percentage points (or 56%) higher likelihood of infections 2 y after the grandchild is born, while in the adjusted model (*H*) this difference is 8.7 percentage points (59%).

### The Role of Geographical Proximity and SES.

**Fig. 4.**
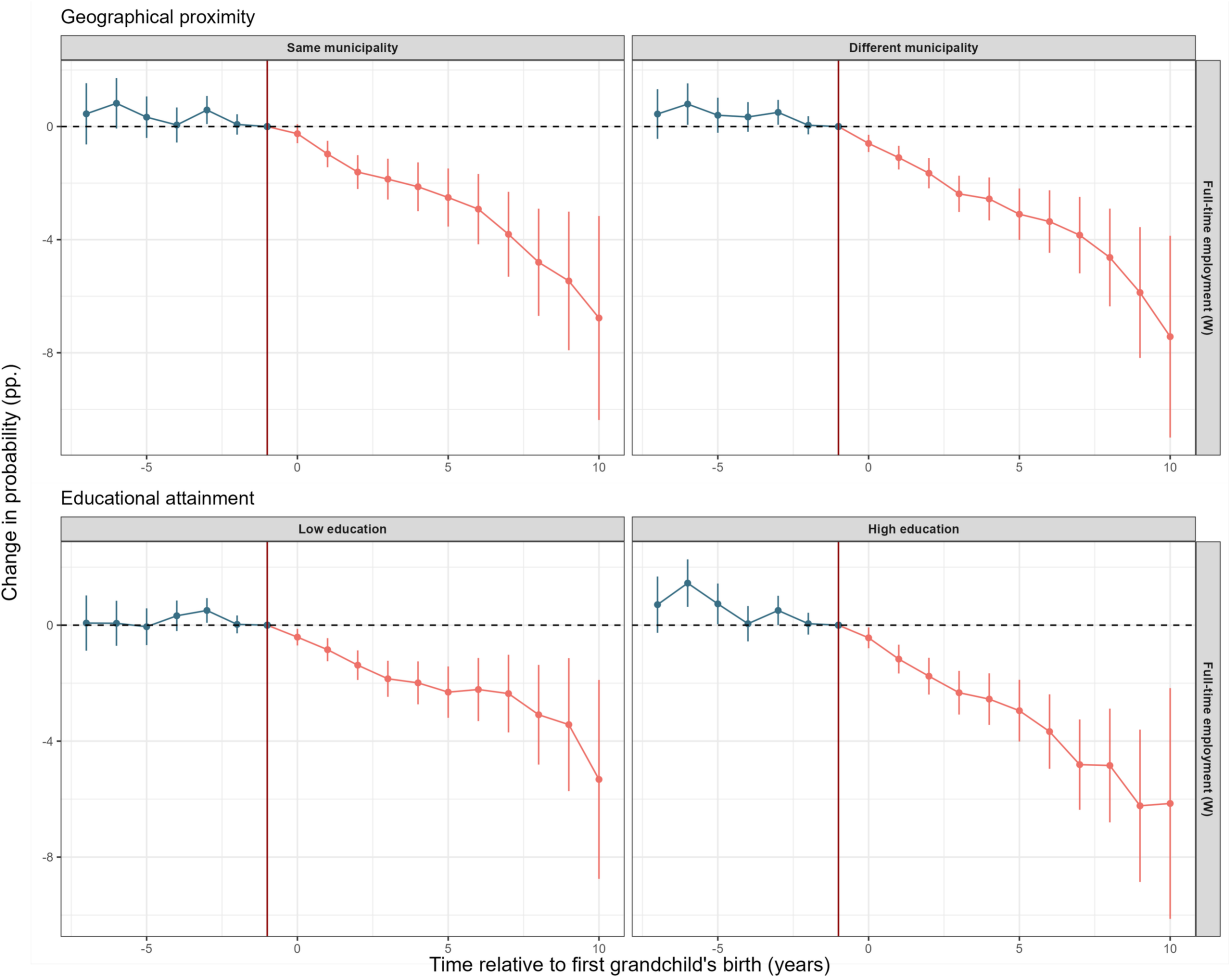
Average treatment effect of the birth of one’s first grandchild (at time 0) on the likelihood of full-time employment for women, by geographical proximity or educational attainment. Geographical distance is defined by whether or not grandparents live in the same municipality as their grandchild. Educational attainment is defined as low for those with no tertiary education, and high for those with tertiary education. The year prior to birth is used as the reference year. Individuals are born in 1950–1960, grandchildren born 2007–2018. Beside individual-specific effects, we control for year and birth year, and those not-yet treated (i.e. not yet grandparents) constitute the control group. Only those with available employment data before and after time −1 are included in this model.

The *Top* panels of Fig. 4 show how the effect of grandparenthood on grandmothers’ full-time employment varies by geographic proximity to their grandchild, specifically whether they live in the same municipality. For both groups, we find a similar decline in full-time employment in the first 5 y of grandparenthood—about 3 percentage points. Ten years after the grandchild’s arrival, the difference in full-time employment is about 7 percentage points. The similar estimates and overlapping CIs between the two conditions mean that geographical distance does not appear to be crucial in terms of neither the direction nor size of estimated effects. We also consider a more fine-grained measure of geographical proximity (neighborhood) and do not find any major differences among the group of grandparents living in the same neighborhood as their grandchild, except for slightly larger effects in terms of respiratory infections (*SI Appendix*, Fig. S15). When we study residence in itself, we also see some interesting patterns. *SI Appendix*, Fig. S12 shows how the likelihood of living in the same municipality as one’s grandchild changes after birth among grandparents who lived in a different municipality the year before birth. There is a clear upward trend, indicating that many of those who originally lived in different municipalities move and end up in the same municipality.^*^

The *Bottom* panels of Fig. 4 show how the likelihood of full-time employment is affected by grandparenthood, depending on the educational attainment of the grandmother. We see a tendency for larger declines in employment among grandmothers with higher educational attainment. However, the overlapping CIs suggest that the effect of grandparenthood on employment does not differ significantly by the grandmother’s educational attainment.

### Age and Health Outcomes.

Last, we consider to what extent being above or below the median age at the transition to grandparenthood^†^ influences estimated health effects. Overall, we find no significant differences by age at transition, with the notable exception of respiratory infections. Among grandparents who transitioned above the median age, the estimated increase in respiratory infections is somewhat larger (*SI Appendix*, Table S6).

## Discussion

This paper has studied the health and labor market consequences of the transition to grandparenthood, focusing on Norwegians who became grandparents for the first time between 2007 and 2018. Leveraging the variation in the timing of the birth of one’s first grandchild, we compared grandparents to not-yet grandparents through an event-study design. We found evidence of both positive and negative health effects as well as clear negative labor market effects. Furthermore, our findings suggest that some of the health effects could be driven by changes in labor market participation. We found that grandparenthood increases the likelihood of respiratory infections, decreases mental, and cardiovascular health issues and does not impact the endocrine or metabolic health of grandparents. Grandparenthood also reduces the likelihood of musculoskeletal health issues among grandmothers, but not among grandfathers. Simultaneously, grandparents respond to the birth of their first grandchild by both working and earning less. The negative effects on full-time employment and earnings were particularly strong for women.

We found that grandparenthood impacts the likelihood of a variety of physical and mental conditions among grandparents. After the arrival of one’s first grandchild, grandparents were significantly more likely to see a doctor due to respiratory conditions. Two years after the grandchild was born, grandmothers saw a 56% increase in the likelihood of seeing their doctor due to such infections, while grandfathers saw a 31% increase in likelihood of visit. We believe that this large gender difference likely reflects the tendency for grandmothers to be more involved in informal childcare provision than grandfathers. This gender gap in grandparental childcare provision is well-documented across Europe (35) and Norway (36). We also found the strongest effect of grandparenthood on infections around 2 to 4 y after birth, coinciding with the period when most children are attending kindergarten and thus are exposed to more bacteria and viruses. While the Norwegian vaccination programme already recommends that those older than 65 take the yearly influenza vaccine, our results highlight why this vaccine might be especially recommended for the 65+ group with young grandchildren.

We also found that grandparents became less likely to consult a doctor for mental and cardiovascular conditions. Over the first 10 y of grandparenthood, consultations for mental disorders declined by 4.5%, and consultations for cardiovascular conditions declined by 3.3%. In addition, grandmothers, but not grandfathers, were 3.8% less likely to consult a doctor for musculoskeletal conditions. Taken together, these patterns suggest improvements not only in mental health, but also in somatic conditions plausibly linked to stress, physical strain, and lifestyle. This is consistent with prior work showing that changes in work intensity and daily routines can affect health (28–31). At the same time, because the estimated effects are not substantially stronger for mental health than for the somatic outcomes, our findings point less to meaning or emotional reward as the main mechanism and more to broader lifestyle-related changes following the transition to grandparenthood. We found no effects on endocrine and metabolic health for either grandparent, in line with expectations. While becoming a grandparent is recognized as a significant social role, to our knowledge there is little research on how this transition in itself is related to changes in health. Previous work has investigated how intergenerational caregiving is associated with outcomes such as mental health and well-being, typically finding that custodial grandparenting is associated with worse health while non-co-residing grandparenting improves grandparental outcomes (12). However, there appears to be significant heterogeneity in effects, as context significantly influences the impact of grandparenthood (37), and most of the previous work on this topic either directly investigates the effect of providing care or investigates the effect of grandparenthood in social settings with a strong emphasis on intergenerational exchange and filial piety. We are also not aware of any work investigating the effect of grandparenting on risk of infections. Consequently, our study improves our understanding of how grandparenthood in itself impacts a variety of health outcomes, and, in particular, in a context where strong welfare state provisions limit the expectations of intergenerational exchange.

Consistent with previous research from the Scandinavian setting (21, 22), we found that grandparenthood significantly impacts both income and labor market participation. On average, our most conservative estimates indicate that men experience an average earnings reduction of about 4 percent, and women around 7 percent, during the first ten years of grandparenthood. However, when accounting for individuals with zero earnings, the magnitude of these effects appears much larger, suggesting that our baseline specification may understate the full earnings effect. We also found a widening of the wage gap within the grandparent-couple after the grandchild was born. However, while both grandmothers and grandfathers saw significant reductions in income, we found a much greater gender difference in the effect of first grandchildren on employment. Toward the end of our observation window, working grandmothers were 7 percentage points, or about 12% less likely to be in full-time employment, while working grandfathers were only about 1.5 percentage points or around 2% less likely to hold a full-time position. This pattern is consistent with the idea that the arrival of grandchildren increases demand for family-provided childcare, and that this demand is met through a gendered reallocation of time within households. In line with theories of household specialization (17), as well as sociological theories emphasizing gendered expectations around care (19), grandmothers appear more likely than grandfathers to adjust labor supply at both the intensive and extensive margin in response to grandparenthood. Our findings also provide further evidence that the gender differences in informal childcare provision among grandparents, as documented in survey data (38), translate into gendered patterns in the labor supply response to grandparenthood. Since transitioning to part-time work late in one’s career can substantially affect pension entitlements, this reduction in labor supply among grandmothers may have longer-term consequences than those captured in this study.

We also examined the extent to which the health and labor market effects of grandparenthood operate independently. Since we find that grandparenthood reduces labor supply, and prior studies suggest that retirement decreases health care utilization (e.g., GP visits) (39), it is plausible that part of the observed health effects are mediated through changes in labor supply. When incorporating both health and labor market effects in the same model, we found evidence of some attenuation of the mental and cardiovascular health effects. In contrast, the effect of grandchildren on respiratory infections and musculoskeletal health did not attenuate when also considering changes in labor supply. Consequently, we believe it is likely that part of the health effects we observe come from the combination of having grandchildren and working less. The notion that reduced labor supply at this margin contributes to improved health, is in line with literature (31).

Finally, we studied whether geographical distance and socioeconomic background influenced the effect of grandparenthood. When comparing grandmothers who lived in the same municipality as their grandchild to those living in different municipalities, we found a decline in the probability of full-time employment in both groups. Consequently, the strong overall employment effects do not appear to be driven solely by grandmothers who, for example, live very close to their grandchildren. In line with previous work, we also found that a significant portion of grandparents move closer to their grandchildren (40, 41). When considering the grandmother’s educational attainment, we also found no statistically significant difference in the employment effect between grandmothers with university education and those with upper secondary education or less. This further supports our interpretation that the observed effects are general rather than driven by specific subgroups of grandparents.

Overall, our findings suggest that the transition to grandparenthood is an important event that reduces the labor market participation of new grandparents and increases the risk of respiratory infections. While the child penalty is well-studied and known, this hidden cost of childcare among grandparents, especially among grandmothers, is less understood. The small but positive effects on mental, musculoskeletal, and cardiovascular health outcomes nuances this picture, and could indicate that grandparenthood positively influences lifestyle and well-being. Whereas grandparents’ decisions to spend time with their grandchildren may be conscious and well-informed, our results highlight an underexplored aspect of childcare costs, that are carried by grandparents and have implications for their health and labor market participation.

While this paper shows how grandchildren significantly impact the lives of first-time grandparents it is not without its limitations. First, while we rely on complete register data for entire birth cohorts, we only observe those who become first-time grandparents in the time window 2007–2018. It is highly likely that the effects of a grandchild are influenced by a variety of contextual factors such as kindergarten and parental leave policies—meaning that grandchildren born outside this specific time window may produce different effects. Second, while primary health care data allow us to study health care utilization, we cannot be certain whether changes in primary health care use reflect changes in health or help-seeking behavior. For example, becoming grandparents may mean that some individuals see their doctor more frequently as they have become more aware of their own health and aging. On the other hand, a combination of lower labor supply and more time spent with family may leave both less of a need and less room to see a doctor due to smaller health niggles. Consequently, this study cannot tell us why grandparents are less likely to see their doctor with mental and cardiovascular health issues.

Nonetheless, our findings have important implications for both public policy and future research. Despite Norway’s extensive childcare services and generous parental support, our results indicate that grandparents continue to play a key role in the provision of childcare. Consequently, policies aimed at addressing gender disparities in the labor market should also take into account the gendered impact of grandparenthood, not just parenthood. Moreover, our study highlights that grandchildren may have multifaceted influences on the health of older adults, warranting further investigation.

## Materials and Methods

### Sample.

Our study population is based on the Norwegian Population Register. This register includes everyone who has been registered as residing in Norway between the 1960 census and data release (2023), and contains information such as gender, date of birth, death, and parent identifiers. This register was linked to educational and socioeconomic register data from 2006 to 2023, and diagnostic data from medical records covering the years 2006–2019. The data from these different sources were joined using pseudonymized individual-specific personal identification numbers. We have limited our analyses to only include individuals who were registered as residing in Norway during the observation period (2006–2019). This ensures that we only use data on individuals who rely on the Norwegian health care system. Individuals who died during our observation window were included until the year of death. Because past research has found grandparenthood effects to depend on time and context, we focused on grandparenthood among individuals born between 1950 and 1960. We chose to focus on these birth cohorts as these are the most relevant birth years among first-time grandparents in our observation window. Furthermore, we also excluded first-generation immigrants and those without complete records of family identifiers as our fertility measure is unreliable for these groups. We limited our analytical sample to those who became first-time grandparents in the window 2007–2018. This ensures we have observed all grandparents both before and after the grandchild’s birth, and also allows us to maximize the use of available data. This leaves us with 2,983,400 observations from 213,100 unique first-time grandparents. Because our estimates rely on staggered treatment timing, this sample contains no separate never-treated control group. Instead, individuals who have not yet had their first grandchild serve as controls until their own transition to grandparenthood, after which they contribute treated observations. For this reason, the control group is best understood not as a distinct set of people, but as the subset of person-year observations belonging to eventual grandparents before treatment. Distributions of age at first grandchild are shown in *SI Appendix*, Fig. S1.

### Measures.

#### Grandparenthood and age at birth of first grandchild.

We use data from the population registry to find grandchildren by first identifying children, and then identifying their children’s children. We only consider legal grandchildren and not other forms of grandparenthood (e.g. step-grandchildren). We calculated the age at grandparenthood in whole years using the birth years of the grandparent and the first grandchild. We use data on grandparenthood covering the period 2006–2023.

#### Health outcomes.

Information on primary health care utilization was obtained from the Norwegian Control and Payment of Health Reimbursements Database (KUHR), established in 2006. We have health data available for the period 2006–2019. The KUHR database contains information from different contact points within the health care system, with the majority of the data coming from primary care physicians. When practitioners submit their reimbursement claims to the Norwegian Health Economics Administration (Helfo), they use the International Classification of Primary Care (ICPC-2) system to register the reason for a patient’s visit (45). We focused on health out- comes which could be influenced by lifestyle and social context, and generated six binary variables—each focusing on a specific set of ICPC-2 codes:

1. **Respiratory infections**: R71-R83, e.g. influenza, the common cold.
2. **Mental health symptoms**: P01-P29, e.g. feeling of anxiety, stress.
3. **Mental health diagnoses**: P70-P99, e.g. depressive disorder, anxiety disorder.
4. **Cardio-vascular health**: K01-K29, e.g. irregular pulse, swollen ankles/ edema, and K70-K99, e.g. hypertension, atrial fibrillation.
5. **Musculoskeletal health**: L01-L29, e.g. back pain or muscle pain, and L70-L99, e.g. fractures, sprains, or other musculoskeletal injuries.
6. **Endocrine/metabolic health**: T01-T29, e.g. weight loss, weight gain, and T70-T99, e.g. obesity, diabetes T1/T2.

Each of these variables equal 1 if an individual has had at least one visit to primary care services in a given year related to the codes in question.

#### Labor market outcomes.

We used Statistics Norway’s tax registry to identify income and labor market participation. For income, we considered earnings from employment. This includes employee income and net income from self-employment earned during each calendar year. We used the Consumer Price Index (CPI) to adjust for inflation, using 2015 as the baseline year. We considered both absolute income (in NOK) and income (in logs). As log-transformations will drop observations with zero income, we also report our results for alternative specifications that allow for zeros: log(income+1) and the inverse hyperbolic sine transformation of income. While income is not a perfect measure of work hours, it is reasonable to assume that within-person changes in income reflect variations in labor supply. To study labor market participation, we used Statistics Norway’s employment data. We looked at all formal employment registered per individual per year and created a new variable called “full-time.” This variable measures whether the individual had at least one full-time position (1) or not (0) in a given year. While some individuals may have multiple part-time positions which add up to full-time employment, we only considered whether one is employed in at least one role classified as full-time employment or not. Importantly, while we have income data available for everyone, data on full-time vs. part-time employment is only available for those with formal employment. We have not interpreted missing employment data as unemployment or retirement and followingly, analysis based on these data restricts the sample to employed grandparents. We focused our analysis of grandparenthood and labor market outcomes on the period 2006–2019 for two reasons: First, it matches the data availability for the health outcomes, and second, it seems plausible that grandparenthood may impact labor market outcomes differently among those who became first-time grandparents during the COVID-19 pandemic. Consequently, we believe the 2020–2023 period should be analyzed separately.

### Covariates.

#### Educational attainment.

Data on educational attainment were obtained from Norway’s National Education Database (NUDB), operated by Statistics Norway. The Norwegian Standard Classification of Education (NUS2000) was used to group individuals based on their highest level of completed education. We grouped individuals into three categories: lower secondary schooling or less (i.e. compulsory education), upper secondary schooling, and university education (i.e. bachelor’s, master’s, and higher). We used educational attainment at the start of our observation period (2006), as it is rare to obtain further education after age 45.

#### Marital status.

We used data from the Norwegian Central Population Register to identify the grandparents’ partnership status. We only considered marriage vs. nonmarriage, as marriage is the most common form of formalized cohabitation in the analyzed birth cohorts.

#### Distance living from grandchildren.

We used data from the Norwegian Central Population Register to identify the mother of the first grandchild’s municipality of residence. Together with information from the same register on grandparents’ municipality of residence, we created a binary variable capturing whether grandparents and grandchildren (likely) lived in the same municipality (1) or not (0) at the time of birth. We used the registered residence in the year before birth to avoid endogeneity (i.e. grandparents moving closer to the grandchildren after birth). We used the mothers’ place of residence as the vast majority of children live with their mother after birth.

#### Demographics.

We also used the population register for the covariates: age, sex, and immigration background. Age is a continuous variable, sex is coded as a binary variable, and immigration background is coded according to an individual’s own and their parents’ country of birth. Those with non-Norwegian parents are grouped into two binary variables: one foreign-born parent or two foreign-born parents. However, as the vast majority of our sample (1950–1960 birth cohorts) are Norwegian-borns with no immigration background (approximately 95%), we do not consider immigration background to be of specific interest in our study population.

### Ethics.

This study was approved by the Regional Committees for Medical and Health Research Ethics (REK) in Norway (#2018/434).

### Statistical Analysis.

#### Descriptive statistics.

In order to look at overall differences between those with and without grandchildren, we present summary statistics for key variables in Table 1. These statistics includes variables such as income, use of health services, and educational attainment.

#### Causal estimates.

To estimate the causal effect of grandparenthood we use an event-study design with not-yet treated as controls. This is the staggered difference-in-difference (DiD) method as seen in Callaway and Sant’Anna (42, 43). We estimated models for grandmothers and grandfathers separately. We also stratified analyses by educational attainment and whether grandparents and grandchildren lived in the same municipality.

We estimated linear probability models using the following specification:

[1] $$\begin{array}{c} Y_{\mathrm{it}}=\sum_{j=-7}^{9}\alpha_{j}\cdot D_{i,t-j}+\gamma_{i}+\lambda_{t}+\beta \cdot X_{\mathrm{it}}+\epsilon_{\mathrm{it}} \end{array}$$

Here, $Y_{\mathrm{it}}$ is the outcome (income, employment status, or presence of a health condition) for individual *i* at time *t*. $D_{i,t-j}$ is a set of event time dummies indicating when the first grandchild’s birth took place relative to the given observation’s calendar time. The coefficient $\alpha_{j}$ captures the dynamic effects of the treatment after the birth has occurred ($\alpha_{j}$ for j≥0). The terms $\alpha_{j}$ for before the birth has occurred (j<0) act as a falsification test. If we assume no anticipation effects, these pre-event terms should not have a trend in *j*. We exclude the event time dummy for t=−1. Consequently, the estimated coefficients measure the impact of grandparenthood relative to this time period. $\gamma_{i}$ represents the individual fixed effects, while $\lambda_{t}$ represents time fixed effects. $X_{\mathrm{it}}$ is a vector of controls for grandparents’ year of birth to capture cohort-specific effects. $\epsilon_{\mathrm{it}}$ is the error term. At any time point, those who had not yet had their first grandchild constitute the control group.

As a robustness check, we also estimated the causal effect of grandparenthood through an alternative method: an instrumental variable event model (“IV-event”). This method is described in detail in *SI Appendix*. All statistical analyses were done using Stata MP17 with the csdid (44) commands. We present our results using the staggered DiD-method in the main section of the paper and report our IV-event results in *SI Appendix*.

## Supplementary Material

Appendix 01 (PDF)

## Data Availability

The data used in this study include primary health care records, employment data, demographic information, and educational outcomes for entire cohorts of the Norwegian population. Therefore, the data used in this study is only available by application to the Regional Committees for Medical and Health Research Ethics and the data owners (the Norwegian Directorate of Health and Statistics Norway). The authors cannot share these data with other researchers due to their sensitive nature and potential for identification. However, researchers can contact the authors if they have questions about the data or have overlapping research projects. The code used for data analysis is available via GitHub (46).

## Footnotes

^*^When we look at which generation changes municipality of residence, we see that about 61% of the moves are initiated by the mother of the grandchild, 17% by the grandparent, and 3 % are related with simultaneous moves. The simultaneous moves also capture cases where parents and grandparents have lived in different municipalities which then have been merged to form one single municipality. The remaining changes in municipality are cases in which municipality of residence data become missing for either the mother or the grandparent.

^†^The median age at the transition to grandparenthood was 56 y in our sample.
