# Supplementary material for: The cost of caring: Gendered health and labor market effects of grandparenthood
Source: Proc Natl Acad Sci U S A. 2026 May 29;123(22):e2535409123. doi: 10.1073/pnas.2535409123 (PMC13229307; doi:10.1073/pnas.2535409123)
Supplement: Supplementary file 1 — Appendix 01 (PDF) [file pnas.2535409123.sapp.pdf]

Supporting Information for

The Cost of Caring: Gendered Health and Labour Market Effects of Grandparenthood

Maria Lyster Andersen, Rannveig Kaldager Hart, Hans Fredrik Sunde, Neil Martin Davies, and Fartein Ask Torvik

Maria Lyster Andersen.
E-mail: marialyster.andersen@fhi.no

This PDF file includes:

Figs. S1 to S16
Tables S1 to S6
SI References

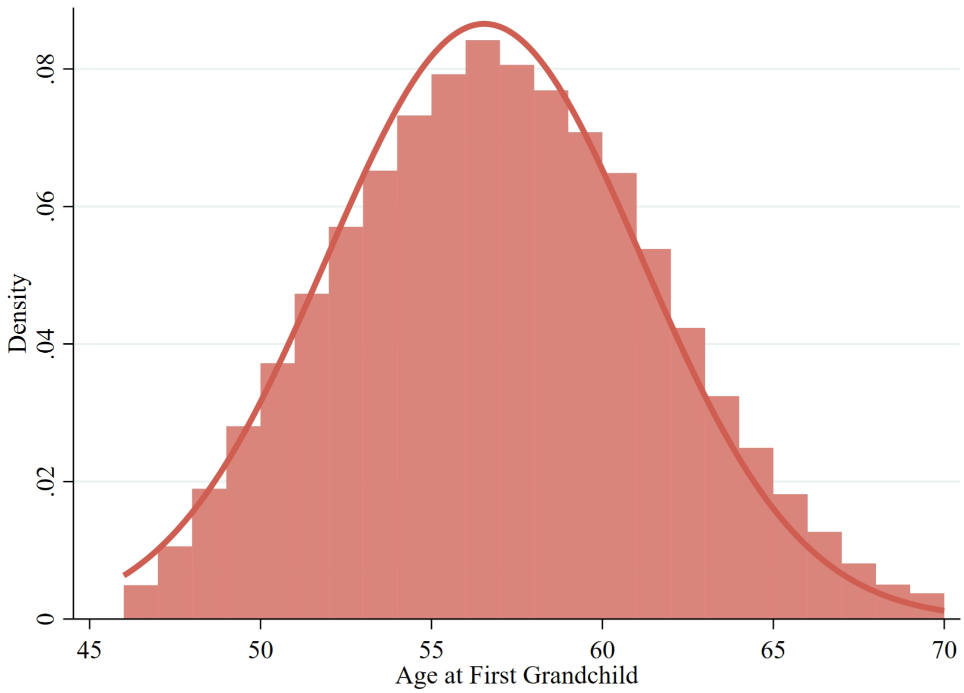

Fig. S1. Distribution of age at first grandchild for women and men (b.1950-1960) when the first grandchild is born in the time window 2006-2019.

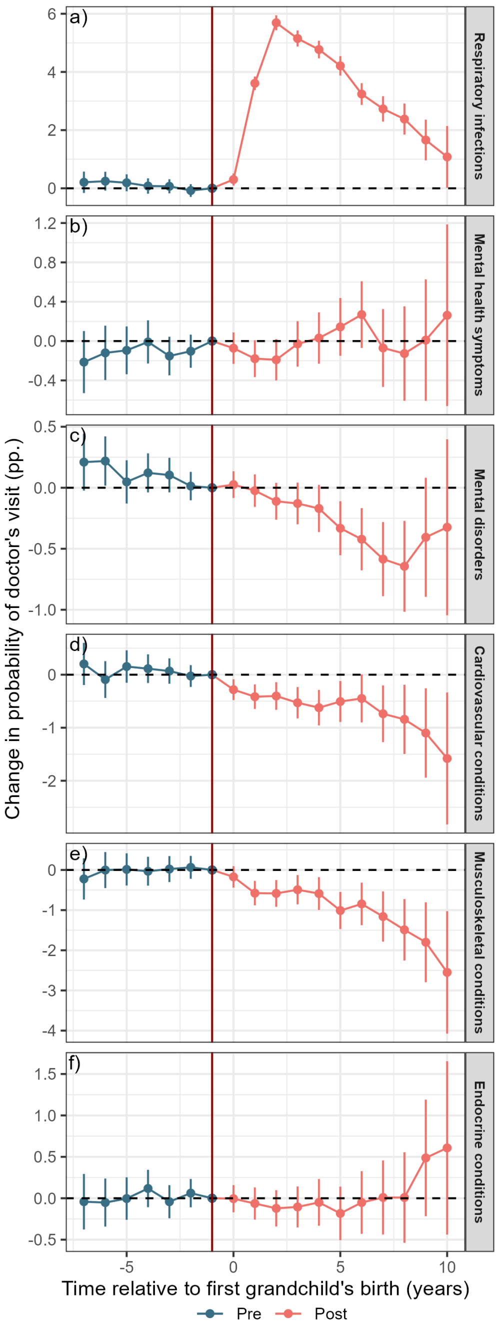

Fig. S2. Average treatment effect of the birth of one's first grandchild (at time 0) on respiratory infections, mental health symptoms, mental disorders, cardiovascular conditions, musculoskeletal conditions, and endocrine conditions. The year before birth is used as the reference year. Individuals are born between 1950-1960, and grandchildren born 2007-2018. Besides individual-specific effects, we control for year and birth year, and those not yet treated (i.e. not yet grandparents) constitute the control group.

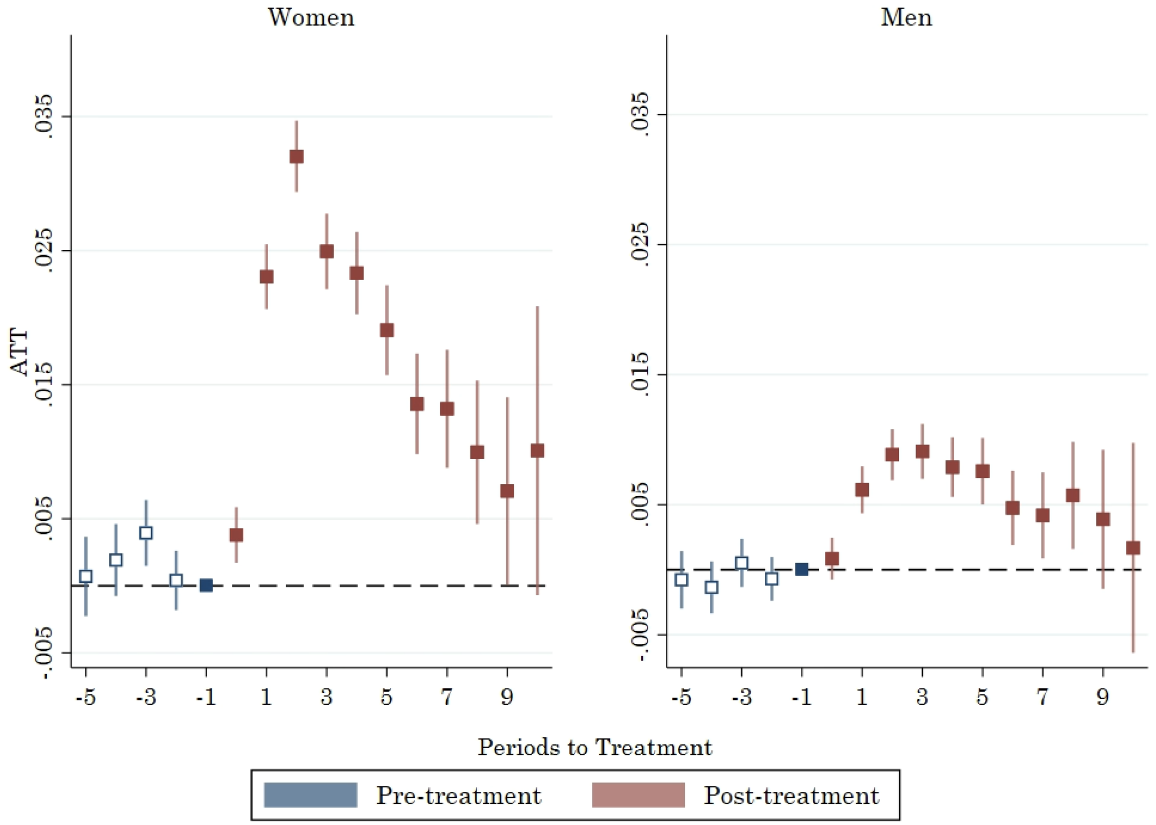

Fig. S3. Average treatment effect of the birth of one's first grandchild (at time 0) on acute upper respiratory infections (typically the common cold). The year before birth is used as the reference year. Individuals are born between 1950-1960, and grandchildren born 2007-2018. Besides individual-specific effects, we control for year and birth year, and those not yet treated (i.e. not yet grandparents) constitute the control group.

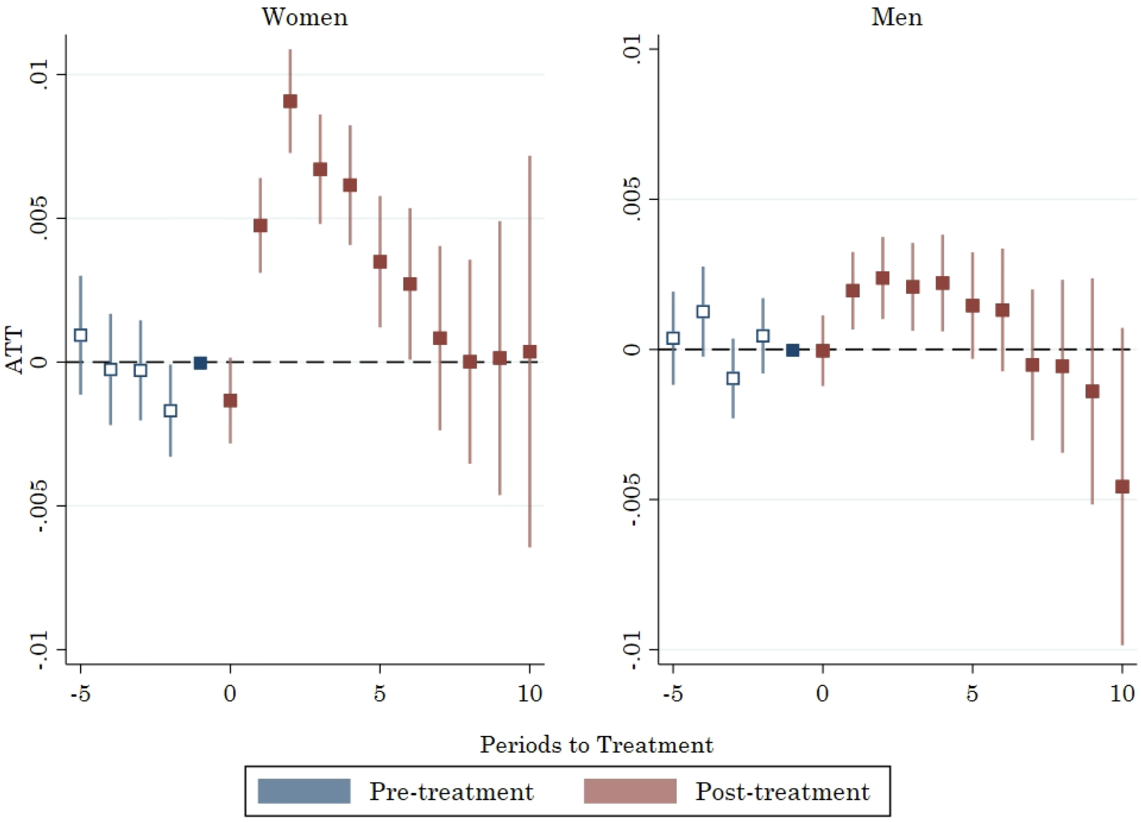

Fig. S4. Average treatment effect of the birth of one's first grandchild (at time 0) on influenza. The year before birth is used as the reference year. Individuals are born between 1950-1960, and grandchildren born 2007-2018. Besides individual-specific effects, we control for year and birth year, and those not yet treated (i.e. not yet grandparents) constitute the control group.

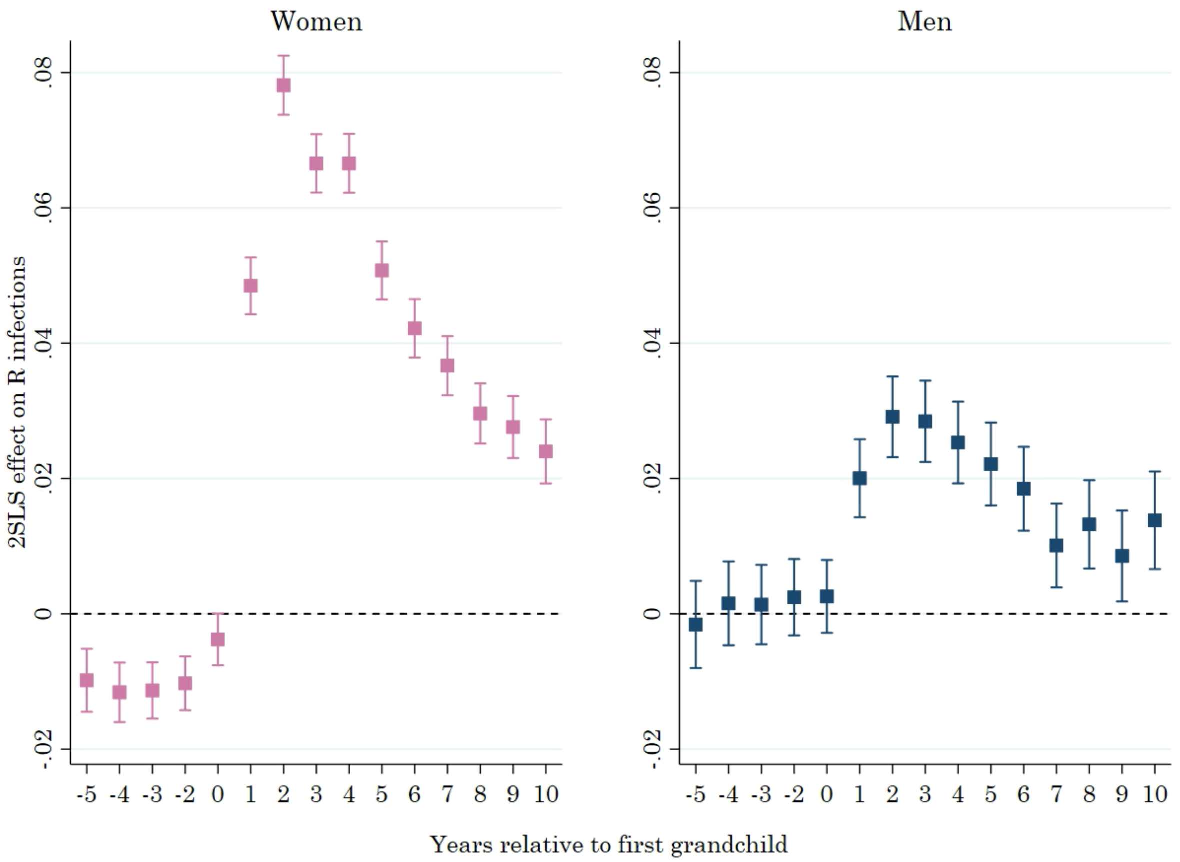

Fig. S5. IV-event estimates of the effect of having a first grandchild on respiratory infections. The year prior to birth is used as the reference year. Individuals are born in 1950-1960. Models includes controls for year, birth year, marital status, immigration background, and educational attainment.

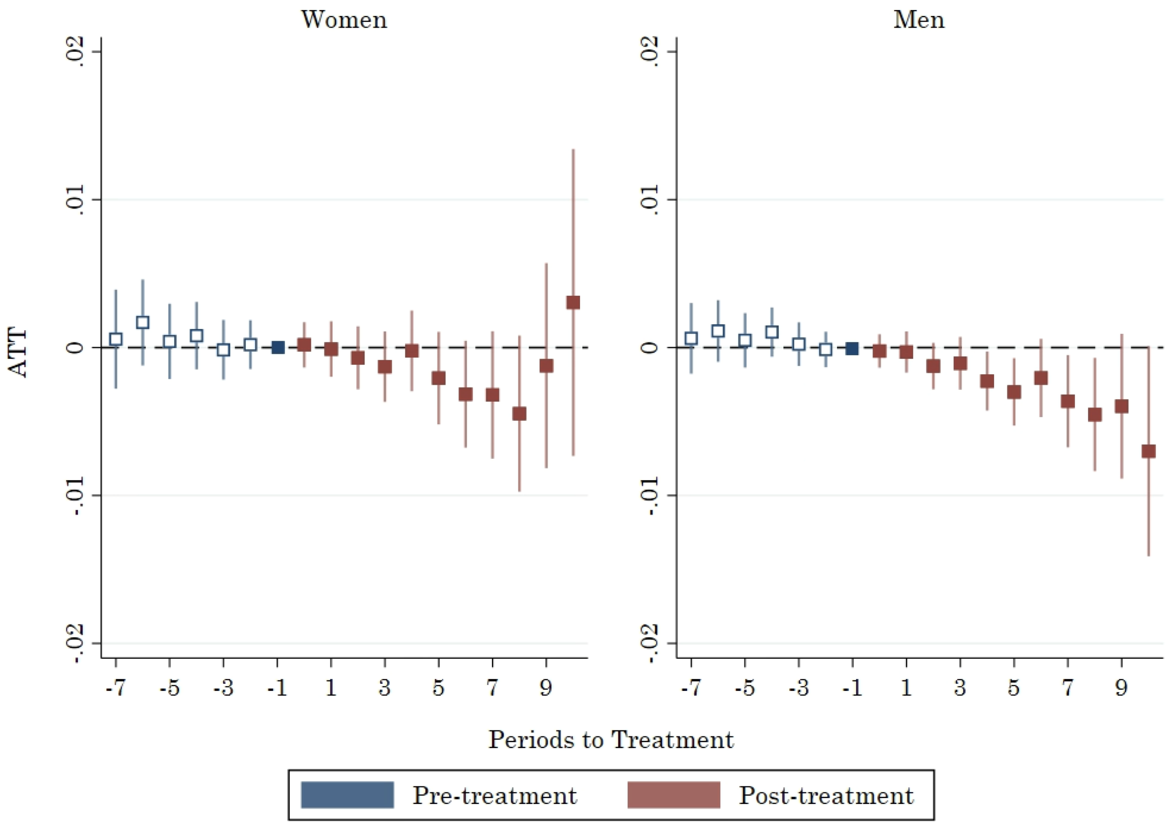

Fig. S6. Average treatment effect of the birth of one's first grandchild (at time 0) on the likelihood of having a depressive disorder for women and men. The year before birth is used as the reference year. Grandparents are born in 1950-1960, grandchildren born 2007-2018. Beside individual-specific effects, we control for year and birth year, and those not-yet treated (i.e. not yet grandparents) constitute the control group.

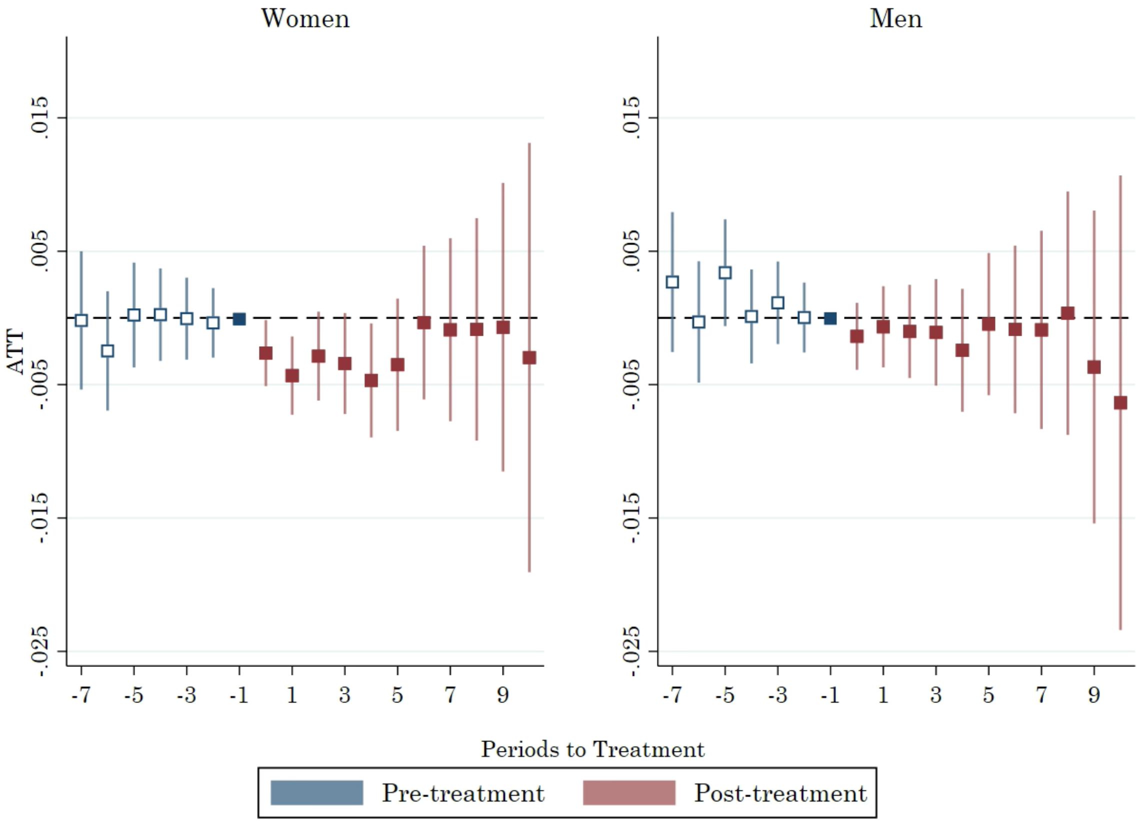

Fig. S7. Average treatment effect of the birth of one's first grandchild (at time 0) on the likelihood of having cardiovascular disease for women and men. The year prior to birth is used as the reference year. Individuals are born in 1950-1960, grandchildren born 2007-2018. Beside individual-specific effects, we control for year and birth year, and those not-yet treated (i.e. not yet grandparents) constitute the control group.

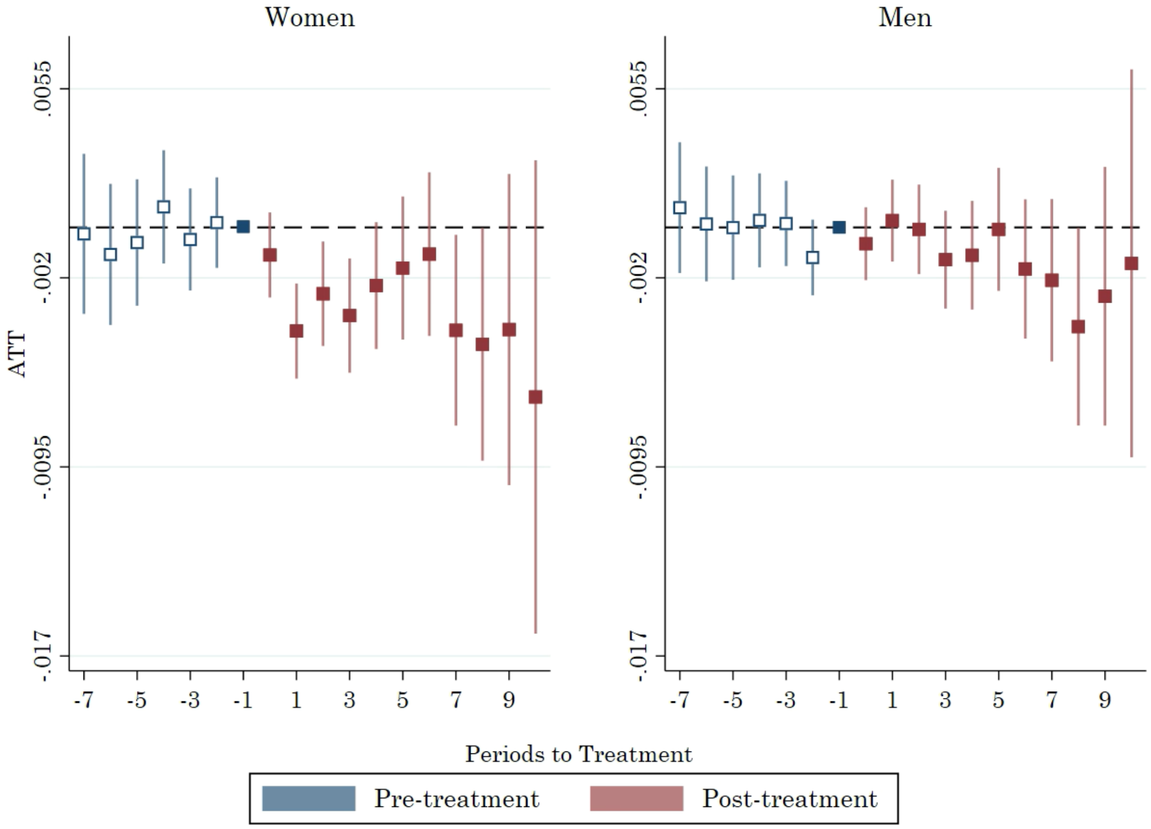

Fig. S8. Average treatment effect of the birth of one's first grandchild (at time 0) on the likelihood of having symptoms related to cardiovascular disease for women and men. The year prior to birth is used as the reference year. Individuals are born in 1950-1960, grandchildren born 2007-2018. Beside individual-specific effects, we control for year and birth year, and those not-yet treated (i.e. not yet grandparents) constitute the control group.

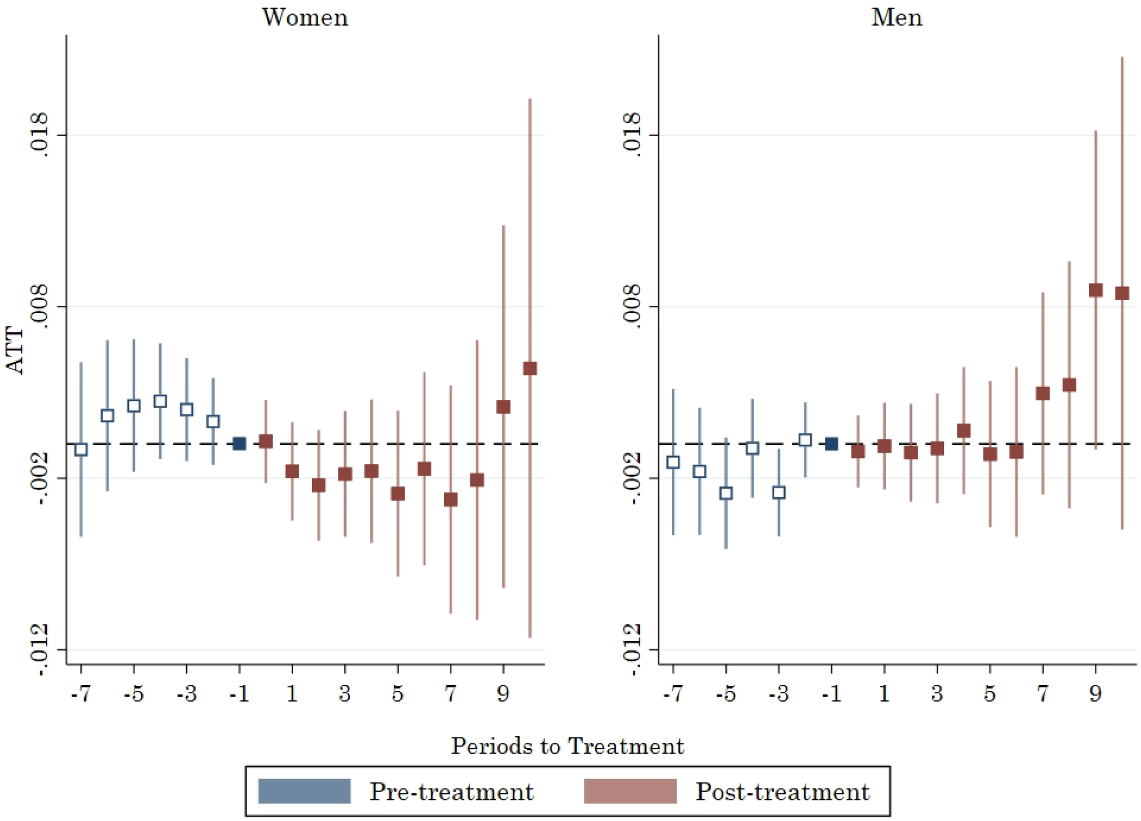

Fig. S9. Average treatment effect of the birth of one's first grandchild (at time 0) on the likelihood of having an endocrine disease for women and men. The year prior to birth is used as the reference year. Individuals are born in 1950-1960, grandchildren born 2007-2018. Beside individual-specific effects, we control for year and birth year, and those not-yet treated (i.e. not yet grandparents) constitute the control group.

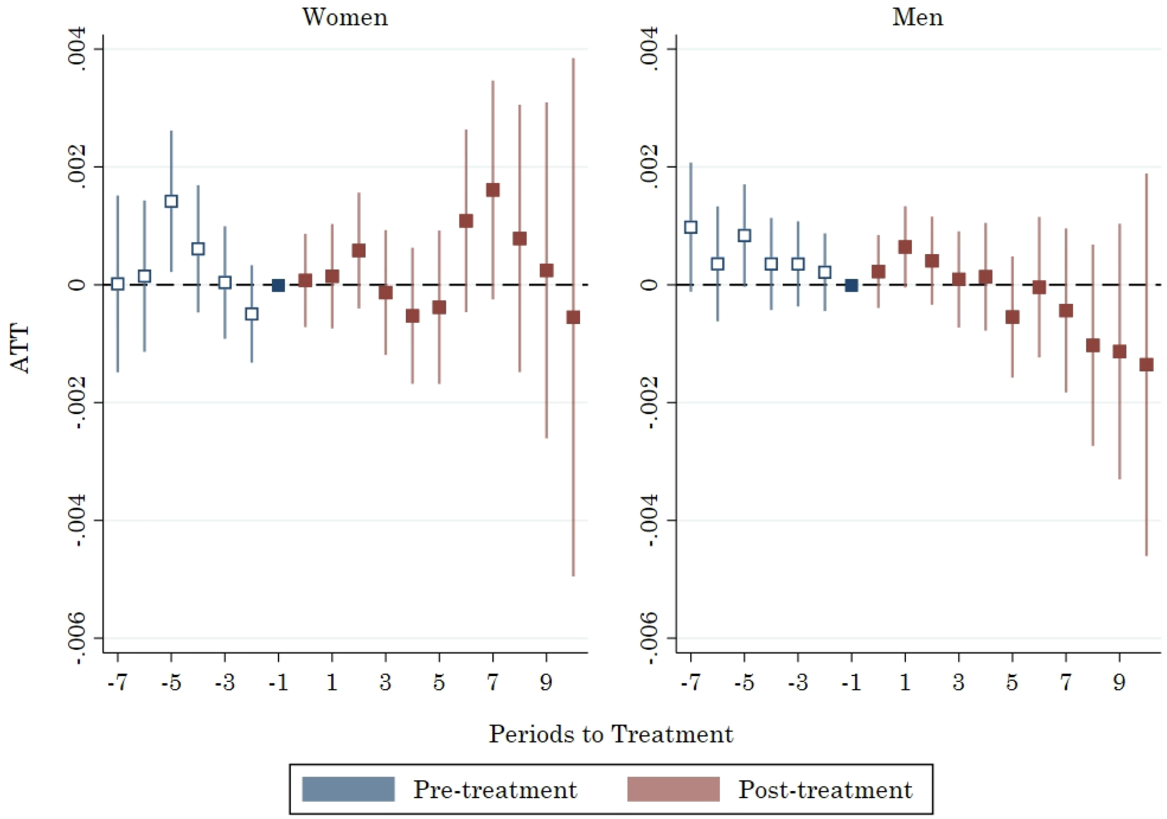

Fig. S10. Average treatment effect of the birth of one's first grandchild (at time 0) on the likelihood of having symptoms related to endocrine conditions for women and men. The year prior to birth is used as the reference year. Individuals are born in 1950-1960, grandchildren born 2007-2018. Beside individual-specific effects, we control for year and birth year, and those not-yet treated (i.e. not yet grandparents) constitute the control group.

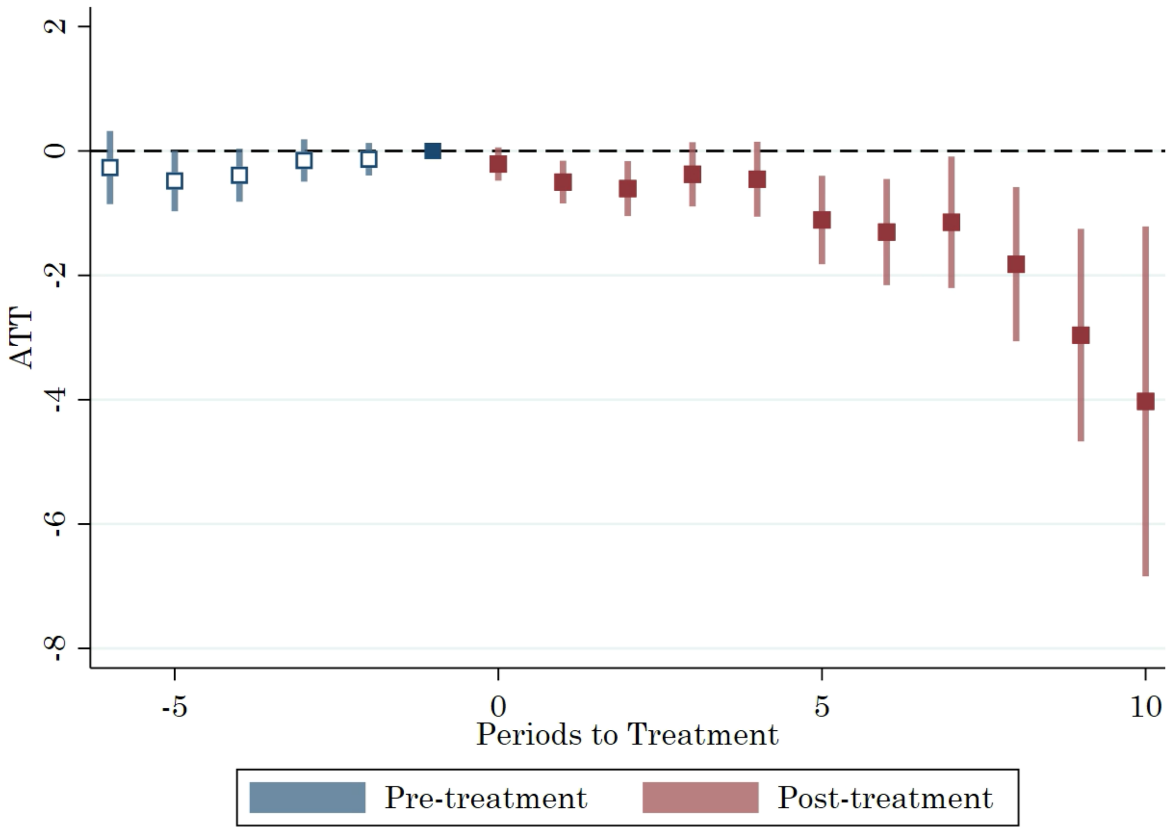

Fig. S11. Average treatment effect of the birth of one's first grandchild (at time 0) on the wage gap within the grandparent-couple (100 * female earnings/male earnings). Grandparents are born in 1950-1960, grandchildren born 2007-2018. Beside individual-specific effects, we control for year and birth year, and those not-yet treated (i.e. not yet grandparents) constitute the control group. At t=-1, the average grandmother's income is 80.6% of the grandfather's income.

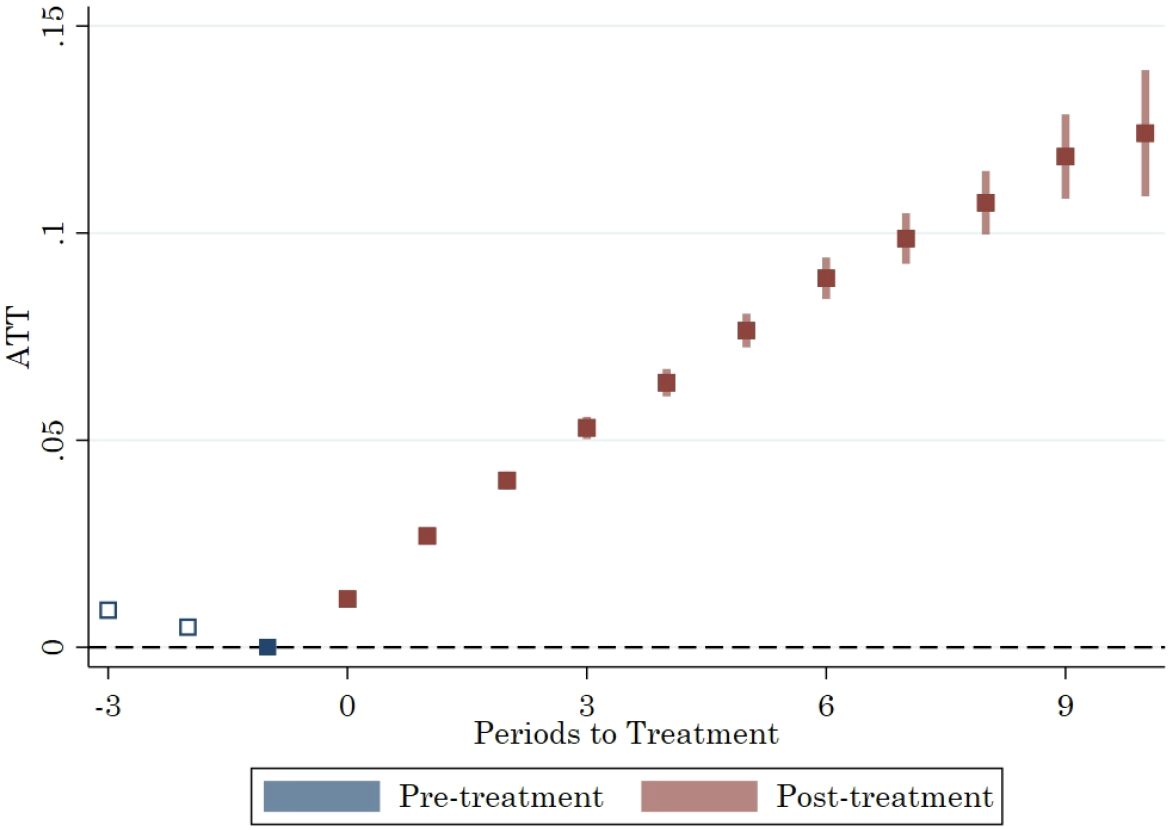

Fig. S12. Average treatment effect of the birth of one's first grandchild (at time 0) on the likelihood of living in the same municipality as one's daughter/daughter-in-law given that one did not live in the same municipality in the year prior to birth. Grandparents are born in 1950-1960, grandchildren born 2007-2018. Beside individual-specific effects, we control for year and birth year, and those not-yet treated (i.e. not yet grandparents) constitute the control group.

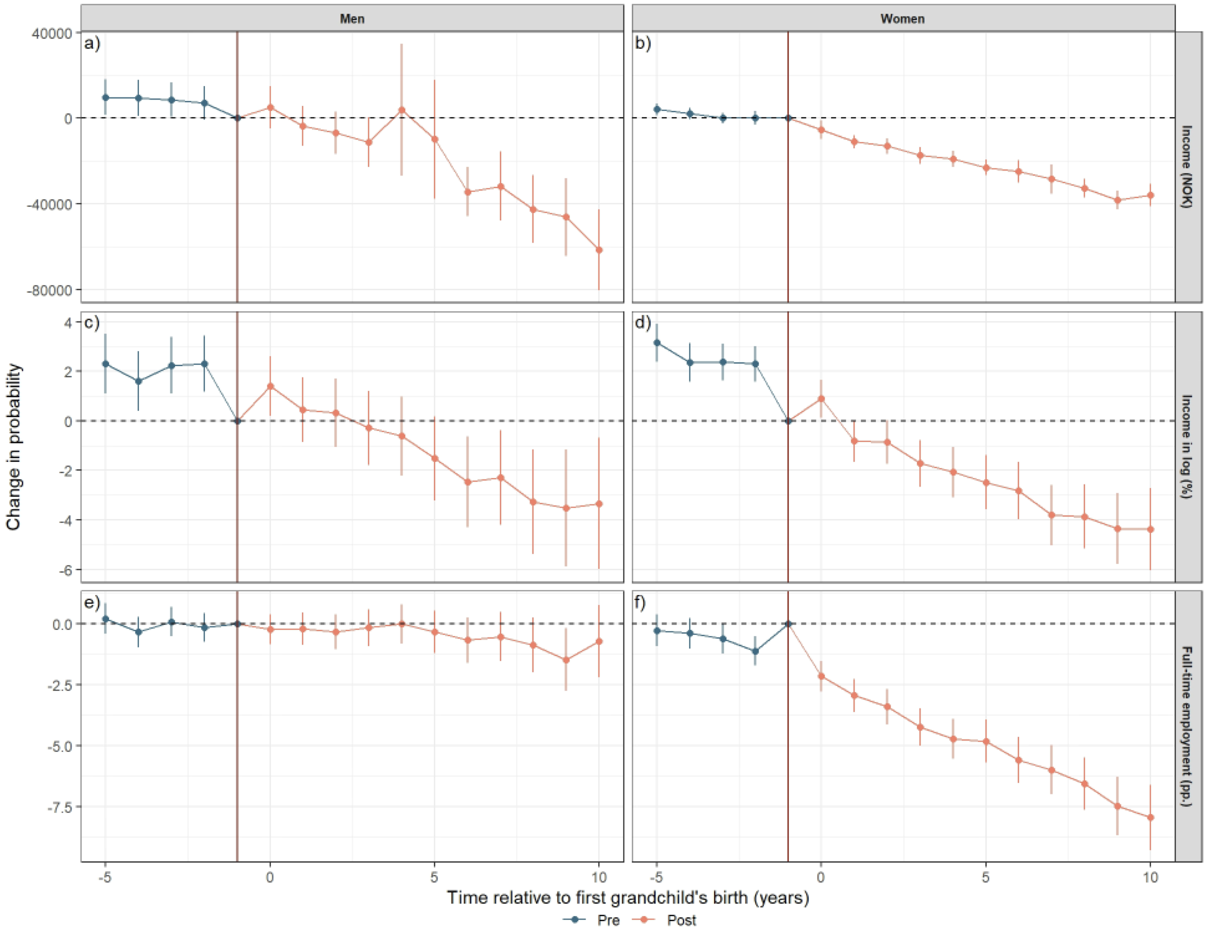

Fig. S13. IV-event estimates of the effect of having a first grandchild on income (in NOK), income (in log), and full-time employment. The instrument is the sex of the grandparents' first-born child. The year prior to birth is used as the reference year. Individuals are born in 1950-1960. Models includes controls for year, birth year, marital status, immigration background, and educational attainment.

Table S1. Health outcomes by gender (part 1)

	Respiratory infections		Musculoskeletal conditions		Psychological symptoms	
	W	M	W	M	W	M
Pre-average	0.00316	-0.000462	-0.00276	0.00230	-0.00165	-0.000506
	(0.00167)	(0.00134)	(0.00214)	(0.00198)	(0.00148)	(0.00113)
Post-average	0.0454***	0.0189***	-0.0176***	-0.00483	0.000433	-0.000813
	(0.00238)	(0.00194)	(0.00330)	(0.00299)	(0.00226)	(0.00172)
Time: -7	0.00665*	-0.00171	-0.00256	-0.00138	-0.00180	-0.00216
	(0.00298)	(0.00236)	(0.00392)	(0.00352)	(0.00265)	(0.00197)
Time: -6	0.00544*	-0.0000457	-0.00232	0.00242	-0.00111	-0.00105
	(0.00263)	(0.00209)	(0.00341)	(0.00310)	(0.00232)	(0.00172)
Time: -5	0.00450	-0.000341	-0.00190	0.00227	-0.000785	-0.000888
	(0.00235)	(0.00186)	(0.00302)	(0.00277)	(0.00204)	(0.00151)
Time: -4	0.000672	0.000940	-0.00538*	0.00445	-0.00208	0.00188
	(0.00212)	(0.00171)	(0.00270)	(0.00250)	(0.00182)	(0.00138)
Time: -3	0.00242	-0.000824	-0.00341	0.00374	-0.00285	-0.000166
	(0.00193)	(0.00156)	(0.00241)	(0.00226)	(0.00162)	(0.00123)
Time: -2	-0.000691	-0.000789	-0.000985	0.00229	-0.00130	-0.000658
	(0.00176)	(0.00142)	(0.00211)	(0.00199)	(0.00138)	(0.00105)
Time: 0	0.00435**	0.00180	-0.00310	-0.000601	-0.00161	-0.0000116
	(0.00165)	(0.00135)	(0.00197)	(0.00188)	(0.00130)	(0.00102)
Time: 1	0.0553***	0.0181***	-0.0104***	-0.00178	-0.00266	-0.00119
	(0.00187)	(0.00152)	(0.00226)	(0.00214)	(0.00152)	(0.00119)
Time: 2	0.0841***	0.0311***	-0.0125***	-0.0000802	-0.00225	-0.00186
	(0.00206)	(0.00165)	(0.00248)	(0.00234)	(0.00170)	(0.00133)
Time: 3	0.0732***	0.0310***	-0.0129***	0.00198	-0.0000951	-0.000866
	(0.00219)	(0.00179)	(0.00272)	(0.00256)	(0.00189)	(0.00147)
Time: 4	0.0672***	0.0290***	-0.0121***	-0.000941	0.000681	-0.000588
	(0.00241)	(0.00195)	(0.00304)	(0.00286)	(0.00213)	(0.00164)
Time: 5	0.0576***	0.0270***	-0.0185***	-0.00362	0.000694	0.00159
	(0.00265)	(0.00217)	(0.00344)	(0.00323)	(0.00242)	(0.00187)
Time: 6	0.0428***	0.0224***	-0.0165***	-0.00256	0.00153	0.00305
	(0.00298)	(0.00246)	(0.00399)	(0.00371)	(0.00277)	(0.00217)
Time: 7	0.0359***	0.0189***	-0.0187***	-0.00644	-0.00174	-0.000530
	(0.00349)	(0.00287)	(0.00471)	(0.00437)	(0.00326)	(0.00253)
Time: 8	0.0309***	0.0174***	-0.0239***	-0.00887	0.00214	-0.00483
	(0.00426)	(0.00353)	(0.00580)	(0.00532)	(0.00401)	(0.00305)
Time: 9	0.0258***	0.00983*	-0.0306***	-0.00854	0.000559	-0.00113
	(0.00565)	(0.00459)	(0.00767)	(0.00687)	(0.00518)	(0.00391)
Time: 10	0.0219*	0.00151	-0.0343**	-0.0217*	0.00752	-0.00258
	(0.00866)	(0.00693)	(0.0118)	(0.0105)	(0.00777)	(0.00585)

Standard errors in parentheses
* $p < 0.05$, ** $p < 0.01$, *** $p < 0.001$

Table S2. Health outcomes by gender (part 2)

	Mental disorders		Cardiovascular conditions		Endocrine conditions	
	W	M	W	M	W	M
Pre-average	0.00131	0.000999	-0.000953	0.00148	0.00159	-0.00114
	(0.00106)	(0.000793)	(0.00165)	(0.00161)	(0.00149)	(0.00127)
Post-average	-0.00244	-0.00261*	-0.00625*	-0.00353	-0.000907	0.00173
	(0.00180)	(0.00132)	(0.00258)	(0.00270)	(0.00239)	(0.00210)
Time: -7	0.00344	0.000873	-0.000669	0.00333	-0.0000455	-0.000613
	(0.00196)	(0.00144)	(0.00293)	(0.00282)	(0.00266)	(0.00222)
Time: -6	0.00211	0.00213	-0.00288	-0.000133	0.00123	-0.00185
	(0.00170)	(0.00125)	(0.00255)	(0.00245)	(0.00230)	(0.00193)
Time: -5	-0.0000182	0.000772	-0.00116	0.00311	0.00278	-0.00234
	(0.00148)	(0.00110)	(0.00225)	(0.00216)	(0.00202)	(0.00169)
Time: -4	0.000581	0.00166	0.000480	0.00110	0.00260	0.0000886
	(0.00133)	(0.000993)	(0.00199)	(0.00191)	(0.00177)	(0.00151)
Time: -3	0.000970	0.00102	-0.00101	0.00175	0.00203	-0.00252
	(0.00117)	(0.000879)	(0.00177)	(0.00169)	(0.00157)	(0.00133)
Time: -2	0.000767	-0.000465	-0.000477	-0.000288	0.000951	0.000367
	(0.000967)	(0.000719)	(0.00152)	(0.00145)	(0.00133)	(0.00115)
Time: 0	0.000505	0.0000717	-0.00414**	-0.00140	0.000198	-0.000304
	(0.000901)	(0.000684)	(0.00144)	(0.00139)	(0.00128)	(0.00110)
Time: 1	-0.000276	-0.0000986	-0.00765***	-0.000302	-0.00178	0.000382
	(0.00110)	(0.000832)	(0.00169)	(0.00165)	(0.00150)	(0.00131)
Time: 2	-0.000827	-0.00117	-0.00512**	-0.00186	-0.00239	-0.000269
	(0.00124)	(0.000948)	(0.00189)	(0.00188)	(0.00169)	(0.00148)
Time: 3	-0.00102	-0.00116	-0.00664**	-0.00232	-0.00207	-0.000296
	(0.00140)	(0.00108)	(0.00212)	(0.00213)	(0.00191)	(0.00167)
Time: 4	-0.000946	-0.00199	-0.00688**	-0.00304	-0.00202	0.000752
	(0.00160)	(0.00121)	(0.00238)	(0.00245)	(0.00217)	(0.00192)
Time: 5	-0.00348	-0.00260	-0.00537*	-0.00140	-0.00308	-0.000789
	(0.00184)	(0.00138)	(0.00274)	(0.00282)	(0.00251)	(0.00220)
Time: 6	-0.00603**	-0.00198	-0.00158	-0.00273	-0.000919	-0.000243
	(0.00212)	(0.00160)	(0.00317)	(0.00332)	(0.00292)	(0.00257)
Time: 7	-0.00631*	-0.00460*	-0.00547	-0.00364	-0.00256	0.00258
	(0.00253)	(0.00190)	(0.00374)	(0.00392)	(0.00346)	(0.00305)
Time: 8	-0.00674*	-0.00533*	-0.00667	-0.00372	-0.00244	0.00241
	(0.00313)	(0.00234)	(0.00454)	(0.00480)	(0.00424)	(0.00373)
Time: 9	-0.00227	-0.00483	-0.00635	-0.00790	0.00221	0.00772
	(0.00415)	(0.00303)	(0.00594)	(0.00617)	(0.00547)	(0.00480)
Time: 10	0.000574	-0.00503	-0.0129	-0.0106	0.00487	0.00713
	(0.00619)	(0.00444)	(0.00892)	(0.00902)	(0.00820)	(0.00713)

Standard errors in parentheses
$^{*}\ p < 0.05$, $^{**}\ p < 0.01$, $^{***}\ p < 0.001$

Table S3. Health outcomes (pooled models)

	Respiratory infections	Mental health symptoms	Mental disorders	Cardiovascular conditions	Musculoskeletal conditions	Endocrine conditions
Pre-average	0.00119	-0.00115	0.00119	0.000704	-0.000260	0.0000688
	(0.00106)	(0.000913)	(0.000651)	(0.00115)	(0.00146)	(0.000971)
Post-average	0.0317***	0.0000435	-0.00284**	-0.00679***	-0.0102***	0.000485
	(0.00151)	(0.00138)	(0.00109)	(0.00188)	(0.00220)	(0.00157)
Time: -7	0.00207	-0.00214	0.00210	0.00203	-0.00223	-0.000416
	(0.00187)	(0.00161)	(0.00119)	(0.00203)	(0.00262)	(0.00171)
Time: -6	0.00245	-0.00120	0.00219*	-0.000917	-0.0000249	-0.000518
	(0.00165)	(0.00141)	(0.00103)	(0.00177)	(0.00229)	(0.00148)
Time: -5	0.00190	-0.000944	0.000479	0.00154	0.000135	-0.0000364
	(0.00148)	(0.00124)	(0.000904)	(0.00156)	(0.00204)	(0.00131)
Time: -4	0.000785	-0.0000873	0.00122	0.00114	-0.000317	0.00118
	(0.00135)	(0.00112)	(0.000815)	(0.00138)	(0.00183)	(0.00115)
Time: -3	0.000693	-0.00152	0.00104	0.000689	0.000223	-0.000418
	(0.00123)	(0.00100)	(0.000722)	(0.00122)	(0.00165)	(0.00102)
Time: -2	-0.000763	-0.00103	0.000137	-0.000252	0.000652	0.000619
	(0.00112)	(0.000859)	(0.000594)	(0.00105)	(0.00145)	(0.000872)
Time: 0	0.00302**	-0.000725	0.000257	-0.00282**	-0.00172	-0.0000505
	(0.00106)	(0.000816)	(0.000559)	(0.001000)	(0.00136)	(0.000836)
Time: 1	0.0361***	-0.00179	-0.000237	-0.00415***	-0.00577***	-0.000632
	(0.00119)	(0.000955)	(0.000680)	(0.00118)	(0.00155)	(0.000991)
Time: 2	0.0569***	-0.00190	-0.00111	-0.00402**	-0.00584***	-0.00122
	(0.00131)	(0.00107)	(0.000772)	(0.00133)	(0.00170)	(0.00112)
Time: 3	0.0515***	-0.000291	-0.00129	-0.00529***	-0.00492**	-0.00105
	(0.00141)	(0.00118)	(0.000871)	(0.00151)	(0.00186)	(0.00126)
Time: 4	0.0477***	0.000306	-0.00170	-0.00622***	-0.00588**	-0.000500
	(0.00154)	(0.00133)	(0.000987)	(0.00171)	(0.00208)	(0.00144)
Time: 5	0.0421***	0.00144	-0.00332**	-0.00506*	-0.0101***	-0.00183
	(0.00170)	(0.00150)	(0.00113)	(0.00197)	(0.00235)	(0.00165)
Time: 6	0.0324***	0.00269	-0.00422**	-0.00448	-0.00846**	-0.000512
	(0.00191)	(0.00173)	(0.00130)	(0.00231)	(0.00270)	(0.00193)
Time: 7	0.0273***	-0.000704	-0.00585***	-0.00737**	-0.0116***	0.0000925
	(0.00223)	(0.00202)	(0.00155)	(0.00273)	(0.00319)	(0.00228)
Time: 8	0.0238***	-0.00127	-0.00644***	-0.00842*	-0.0149***	0.0000881
	(0.00272)	(0.00245)	(0.00190)	(0.00333)	(0.00390)	(0.00279)
Time: 9	0.0166***	0.0000967	-0.00406	-0.0110*	-0.0180***	0.00487
	(0.00357)	(0.00315)	(0.00249)	(0.00430)	(0.00508)	(0.00359)
Time: 10	0.0108*	0.00262	-0.00324	-0.0158*	-0.0255**	0.00608
	(0.00542)	(0.00471)	(0.00368)	(0.00635)	(0.00778)	(0.00534)

Standard errors in parentheses
$^{*}\ p < 0.05$, $^{**}\ p < 0.01$, $^{***}\ p < 0.001$

Table S4. Labour market outcomes (Part 1)

	Income (CPI adjusted)		Income (log)		Full-time employment	
	W	M	W	M	W	M
Pre-average	2970.9***	1680.7	0.00882**	0.0134***	0.00416*	0.000355
	(672.0)	(1749.8)	(0.00289)	(0.00287)	(0.00175)	(0.00140)
Post-average	-20080.5***	-13788.9***	-0.0677***	-0.0398***	-0.0319***	-0.00858**
	(1370.1)	(3653.8)	(0.00648)	(0.00659)	(0.00357)	(0.00295)
Time: -7	3306.0**	6809.9*	0.00877	0.0206***	0.00463	0.000216
	(1181.3)	(3074.2)	(0.00510)	(0.00499)	(0.00348)	(0.00265)
Time: -6	3835.4***	1396.1	0.00952*	0.0180***	0.00820**	-0.00157
	(1066.5)	(2525.2)	(0.00432)	(0.00421)	(0.00289)	(0.00222)
Time: -5	3539.9***	357.8	0.0127***	0.0142***	0.00383	-0.000493
	(842.4)	(2924.9)	(0.00367)	(0.00359)	(0.00241)	(0.00187)
Time: -4	2659.0***	814.3	0.00707*	0.0124***	0.00228	0.000424
	(689.3)	(1950.5)	(0.00318)	(0.00307)	(0.00204)	(0.00160)
Time: -3	2400.4***	188.3	0.00779**	0.00750**	0.00541**	0.00146
	(594.6)	(1428.5)	(0.00264)	(0.00261)	(0.00167)	(0.00132)
Time: -2	2084.5***	517.8	0.00707***	0.00736***	0.000587	0.00210*
	(629.0)	(1428.5)	(0.00195)	(0.00196)	(0.00122)	(0.000983)
Time: 0	-2225.2***	-2379.3*	-0.00986***	-0.00360	-0.00449***	-0.00113
	(398.2)	(1166.0)	(0.00189)	(0.00193)	(0.00115)	(0.000963)
Time: 1	-6020.5***	-4012.7*	-0.0276***	-0.00773**	-0.0105***	-0.00232
	(580.3)	(1561.2)	(0.00277)	(0.00271)	(0.00159)	(0.00133)
Time: 2	-9275.3***	-5249.5**	-0.0340***	-0.0175***	-0.0165***	-0.00264
	(765.7)	(1917.4)	(0.00331)	(0.00340)	(0.00203)	(0.00169)
Time: 3	-12351.9***	-10028.7***	-0.0395***	-0.0189***	-0.0218***	-0.00447*
	(858.1)	(2268.8)	(0.00400)	(0.00408)	(0.00243)	(0.00206)
Time: 4	-15057.6***	-11229.9**	-0.0488***	-0.0248***	-0.0241***	-0.00538*
	(1069.8)	(3480.4)	(0.00480)	(0.00490)	(0.00289)	(0.00246)
Time: 5	-18973.0***	-13830.0***	-0.0590***	-0.0305***	-0.0288***	-0.00854**
	(1292.5)	(3346.8)	(0.00578)	(0.00589)	(0.00345)	(0.00296)
Time: 6	-23069.5***	-16408.4***	-0.0722***	-0.0416***	-0.0320***	-0.0128***
	(1564.2)	(3911.2)	(0.00704)	(0.00724)	(0.00418)	(0.00360)
Time: 7	-27791.7***	-19054.8***	-0.0956***	-0.0574***	-0.0386***	-0.0151***
	(1943.1)	(4914.6)	(0.00892)	(0.00913)	(0.00508)	(0.00439)
Time: 8	-30840.5***	-18960.3**	-0.102***	-0.0685***	-0.0475***	-0.0145**
	(2392.0)	(6027.3)	(0.0114)	(0.0119)	(0.00644)	(0.00554)
Time: 9	-35242.2***	-26671.0***	-0.118***	-0.0783***	-0.0573***	-0.00945
	(3049.4)	(7750.9)	(0.0161)	(0.0161)	(0.00851)	(0.00746)
Time: 10	-40038.4***	-23853.9	-0.138***	-0.0893***	-0.0695***	-0.0179
	(4331.0)	(12682.8)	(0.0253)	(0.0249)	(0.0128)	(0.0111)

Standard errors in parentheses
$^{*}\ p < 0.05$, $^{**}\ p < 0.01$, $^{***}\ p < 0.001$

Table S5. Labour market outcomes (Part 2)

	Income (log+1)		Income (ihs-trans.)	
	W	M	W	M
Pre-average	0.0336***	0.0183	0.0340**	0.0147
	(0.0105)	(0.00988)	(0.0115)	(0.0110)
Post-average	-0.339***	-0.150***	-0.353***	-0.147***
	(0.0279)	(0.0256)	(0.0297)	(0.0275)
Time: -7	0.0533**	0.0289	0.0548*	0.0167
	(0.0201)	(0.0180)	(0.0220)	(0.0200)
Time: -6	0.0415*	0.0169	0.0422*	0.00297
	(0.0165)	(0.0151)	(0.0181)	(0.0168)
Time: -5	0.0388**	0.0185	0.0371*	0.0157
	(0.0137)	(0.0127)	(0.0150)	(0.0142)
Time: -4	0.0291**	0.0141	0.0297*	0.0158
	(0.0112)	(0.0105)	(0.0124)	(0.0119)
Time: -3	0.0250**	0.0199*	0.0247*	0.0194*
	(0.00882)	(0.00837)	(0.00981)	(0.00967)
Time: - 2	0.0140*	0.0116	0.0154*	0.0176*
	(0.00607)	(0.00609)	(0.00690)	(0.00723)
Time: 0	-0.0166**	-0.00351	-0.0189**	0.00226
	(0.00600)	(0.00603)	(0.00679)	(0.00716)
Time: 1	-0.0560***	-0.0253**	-0.0587***	-0.0271**
	(0.00945)	(0.00911)	(0.0104)	(0.0104)
Time: 2	-0.125***	-0.0562***	-0.129***	-0.0619***
	(0.0127)	(0.0120)	(0.0138)	(0.0134)
Time: 3	-0.182***	-0.0909***	-0.191***	-0.0988***
	(0.0161)	(0.0152)	(0.0174)	(0.0167)
Time: 4	-0.222***	-0.117***	-0.234***	-0.120***
	(0.0198)	(0.0188)	(0.0213)	(0.0205)
Time: 5	-0.287***	-0.148***	-0.296***	-0.151***
	(0.0246)	(0.0230)	(0.0264)	(0.0250)
Time: 6	-0.378***	-0.180***	-0.399***	-0.174***
	(0.0303)	(0.0283)	(0.0324)	(0.0306)
Time: 7	-0.474***	-0.214***	-0.493***	-0.207***
	(0.0379)	(0.0350)	(0.0405)	(0.0377)
Time: 8	-0.574***	-0.251***	-0.591***	-0.243***
	(0.0481)	(0.0228)	(0.0513)	(0.0479)
Time: 9	-0.664***	-0.294***	-0.690***	-0.285***
	(0.0641)	(0.0591)	(0.0684)	(0.0635)
Time: 10	-0.755***	-0.271**	-0.787***	-0.242**
	(0.0957)	(0.0864)	(0.101)	(0.0931)

Standard errors in parentheses
* $p < 0.05$, ** $p < 0.01$, *** $p < 0.001$

Table S6. Employment and respiratory infections: by marital status and age.

	Full-time employment			Respiratory infections	
	Married	Unmarried	Divorced/widowed	Above median age	All ages
Pre-average	0.00370	0.00543	0.00653	0.00132	0.00119
	(0.00209)	(0.00316)	(0.00357)	(0.00138)	(0.00106)
Post-average	-0.0310***	-0.0339***	-0.0302***	0.0599***	0.0317***
	(0.00439)	(0.00617)	(0.00701)	(0.0132)	(0.00151)
Tm7	0.00264	0.00925	0.0112	0.00280	0.00207
	(0.00421)	(0.00615)	(0.00764)	(0.00216)	(0.00187)
Tm6	0.00604	0.0136**	0.0124*	0.00149	0.00245
	(0.00347)	(0.00521)	(0.00593)	(0.00200)	(0.00165)
Tm5	0.00396	0.00387	0.00546	0.00246	0.00190
	(0.00288)	(0.00442)	(0.00500)	(0.00186)	(0.00148)
Tm4	0.00213	0.00303	0.00339	0.000568	0.000785
	(0.00242)	(0.00379)	(0.00425)	(0.00177)	(0.00135)
Tm3	0.00623**	0.00360	0.00535	0.00272	0.000693
	(0.00199)	(0.00310)	(0.00346)	(0.00168)	(0.00123)
Tm2	0.00118	-0.000813	0.00140	0.000848	-0.000763
	(0.00145)	(0.00230)	(0.00255)	(0.00161)	(0.00112)
Tp0	-0.00504***	-0.00311	-0.000849	0.00560*	0.00302**
	(0.00136)	(0.00214)	(0.00235)	(0.00218)	(0.00106)
Tp1	-0.0109***	-0.00949**	-0.00892**	0.0442***	0.0361***
	(0.00189)	(0.00295)	(0.00326)	(0.00282)	(0.00119)
Tp2	-0.0175***	-0.0141***	-0.0108*	0.0766***	0.0569***
	(0.00241)	(0.00377)	(0.00418)	(0.00377)	(0.00131)
Tp3	-0.0226***	-0.0199***	-0.0163**	0.0731***	0.0515***
	(0.00290)	(0.00449)	(0.00497)	(0.00468)	(0.00141)
Tp4	-0.0241***	-0.0243***	-0.0209***	0.0752***	0.0477***
	(0.00345)	(0.00530)	(0.00589)	(0.00616)	(0.00154)
Tp5	-0.0268***	-0.0335***	-0.0309***	0.0716***	0.0421***
	(0.00416)	(0.00622)	(0.00694)	(0.00842)	(0.00170)
Tp6	-0.0310***	-0.0341***	-0.0310***	0.0606***	0.0324***
	(0.00508)	(0.00739)	(0.00828)	(0.0113)	(0.00191)
Tp7	-0.0364***	-0.0433***	-0.0385***	0.0500**	0.0273***
	(0.00621)	(0.00890)	(0.0101)	(0.0155)	(0.00223)
Tp8	-0.0449***	-0.0542***	-0.0485***	0.0865***	0.0238***
	(0.00786)	(0.0113)	(0.0130)	(0.0242)	(0.00272)
Tp9	-0.0561***	-0.0604***	-0.0474**	0.0609	0.0166***
	(0.0105)	(0.0147)	(0.0169)	(0.0379)	(0.00357)
Tp10	-0.0662***	-0.0768***	-0.0785**	0.0543	0.0108*
	(0.0155)	(0.0227)	(0.0259)	(0.0727)	(0.00542)

For full-time employment, the sample is restricted to grandmothers only. Standard errors in parentheses
* $p < 0.05$, ** $p < 0.01$, *** $p < 0.001$

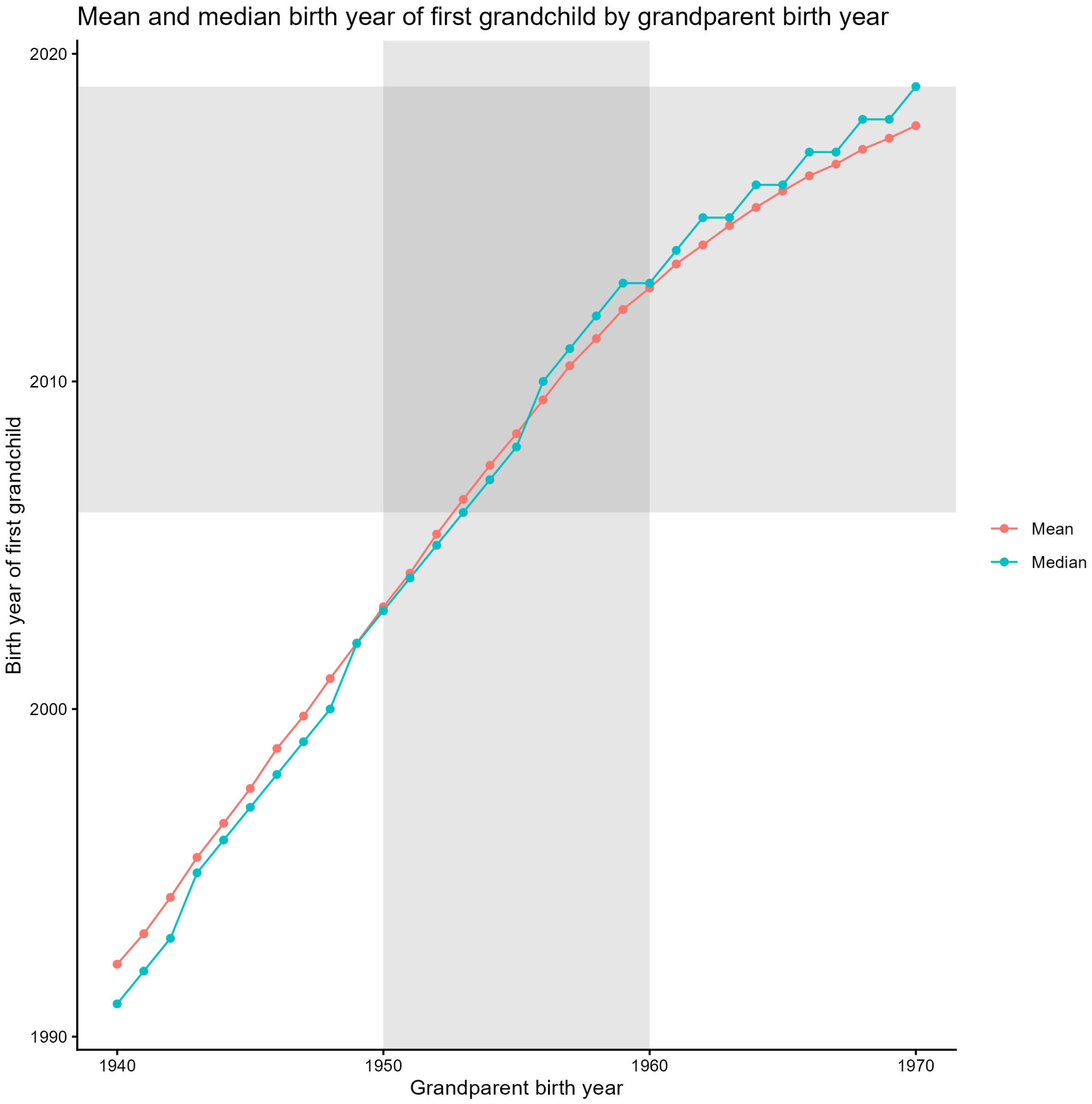

Fig. S14. The mean and median birth year of the first grandchild by the grandparents' own birth cohort. Calculated based on birth records until 2024, which means that especially the later cohorts will have higher mean and medians once more birth records are included. Shaded y-axis represent the observation years of this paper, shaded x-axis represent the birth cohorts studied in the paper.

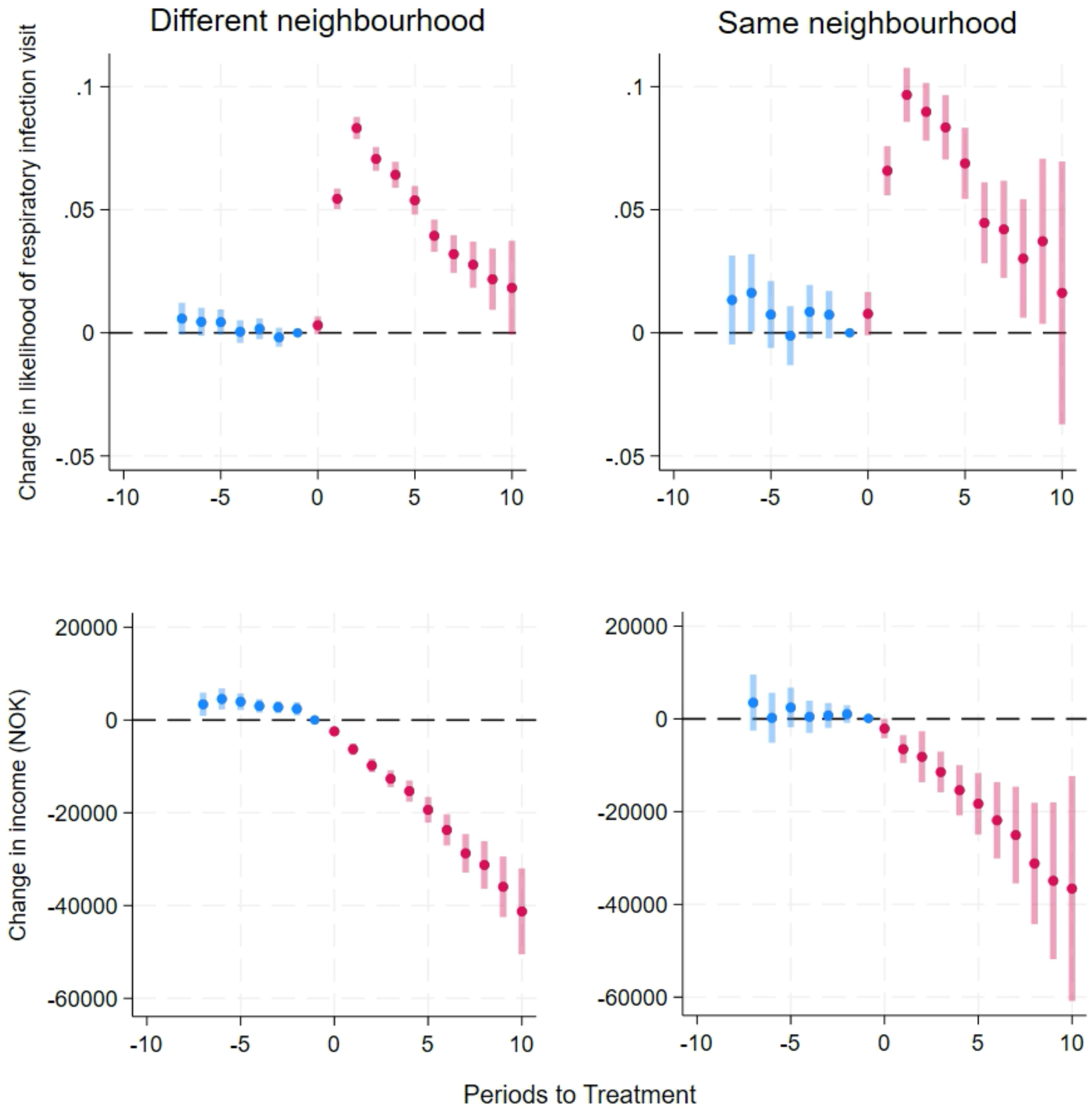

Fig. S15. Average treatment effect of the birth of one's first grandchild (at time 0) income (NOK) and respiratory infections. The sample restricted to grandmothers only, and the analysis is stratified by whether grandparents and the mother of the unborn grandchild lived in the same neighbourhood the year before birth. The year prior to birth is used as the reference year. Individuals are born in 1950-1960, grandchildren born 2007-2018. Beside individual-specific effects, we control for year and birth year, and those not-yet treated (i.e. not yet grandparents) constitute the control group.

IV-Event

We use the gender of the grandparent's first-born child to find random variation in the timing of grandparenthood. Parents of first-born girls are more likely to become grandparents earlier, and we use this fact to find exogenous variation in the likelihood of having a grandchild of age ψ. However, as women typically provide more care for their parents (1), being the parent of a first-born girl could be beneficial in terms of certain life-style related health outcomes – regardless of grandparenthood. For example, one can imagine that better care and more frequent follow-up from a daughter could be related to hypertension risk. However, we do not consider it likely that having a daughter as opposed to a son meaningfully impacts the risk of respiratory infections throughout the year. Consequently, we only use this IV-event method for labour market outcomes and respiratory infections.

To estimate our IV-event models we used the following specifications:

$$GrP_{age\,grandchild_{it}=\psi} = \pi_0 + \pi_1 Z_i + \pi_\psi \left(Z_i \times \mathbf{1}\{\text{event time}_{it} = \psi\}\right) + \pi_3 W_i + \nu_i, \psi \in \{-5, -4, \ldots, 10\} \quad [1]$$

$$Y_{it} = \beta_0 + \sum_{\psi \geq 0} \delta_\psi \widehat{GrP_{age\,grandchild_{it}=\psi}} + \beta_2 W_i + \epsilon_i \quad [2]$$

Equation 1 is the first step and equation 2 is the second step of the 2SLS estimation. $GrP_{age\,grandchild_{it}=\psi}$ is binary variable equal to 1 if individual i at time t has a ψ-year-old grandchild. Z_i is our instrument and a binary variable equal to 1 if the first-born child of individual i is a girl. W_i is a vector of controls (education, marital status, ethnicity/immigration background), and v_i and ϵ_i are error terms. Y_{it} is our outcome (income or labour market participation) and δ_ψ is our main coefficient of interest estimating the dynamic effect of grandparenthood at $\psi \geq 0$. Similarly to our event-study, we omit $\psi = -1$ from our models to use the year prior to grandparenthood as the reference year. While our event-study by design limits our sample to only those who eventually become grandparent, we do not make this same restriction in the IV-event design. Limiting our 2SLS sample similarly to the event design would implicitly condition on the instrument outcome and therefore violate instrument validity. However, in order to look at a similar sample in the IV-event analysis and event-study, we limit our 2SLS sample to those with first-borns born in the time-window 1980-1990 (inclusive), which is the most common birth years of parents to the grandchildren born 2007-2018. So while restricting our sample to only those with grandchildren born in 2007-2018 would violate validity as our instrument influences the likelihood of being in this sample, there is no possibility that our instrument influences the likelihood of being in the sample of parents to children born between 1980 and 1990. This leaves a sample size similar to our event-study: in total 182,542 unique individuals.

Notes about the local average treatment effect (LATE): Previous research has shown that maternal grandparents often live closer to their children (2), and tend to be more involved than paternal grandparents (3). Furthermore, there is also some evidence suggesting that having daughters rather than sons is beneficial for parents' health at older ages (4). Daughters may also be more likely to nudge their parent to see a doctor in case of health issues compared to sons. For these reasons, we believe that our instrument is unsuitable in the analysis of most of our health outcomes, with the exception of respiratory infections. Therefore, we will only use the IV-event analysis for labour market outcomes and respiratory infections. It is also possible that even for labour market outcomes, these considerations could be reflected in the estimated effect sizes of grandparenthood.

Assumption checks for the instrument:

1. *Relevance and monotonicity:* When we look at the distribution of grandparents' age at the birth of their first grandchild for those with a first-born girl compared to a first-born boy, we see that both men and women shift to the left (Fig. S16). This means that both men and women are affected by their first-born's sex. We also find that the shifting occurs across all ages. A first stage regression of how the first-born's sex predicts grandparenthood also reveals a strongly relevant instrument ($\beta = 0.05$, F-stat of excluded IV = 76.7).

2. *Exclusion restriction and exogeneity:* One could imagine that the sex of one's first born child impacts other characteristics influencing labour market outcomes. For example, the first born's sex could impact fertility or relationship stability, which in turn could affect a person's income. Testing this, we find that parents of first-born girls on average became grandparents earlier (1.5 years for women and 1.2 years for men), but that the gender of the first born child was not significantly/meaningfully related to fertility or divorce. Approximately 81.4% of those with a first-born girl went on to have a second child, compared to 81.6% among those with a first-born boy. While this difference is statistically significant, the magnitude of the difference is so small that we do not consider the difference as meaningful. Divorce was equally common at 20.4% among those with a first-born girl or a first-born boy ($p = 0.97$).

3. *Independence:* We consider the sex of a child as randomly determined at conception. While one could expect sex-selective abortions in certain settings, we consider this as unlikely to happen in Norway. Self-determined abortion was only available up to week 12 in Norway, and the sex of the fetus can usually first be determined reliably after week 12 (5). When looking at income, we also found no significant difference between the two groups (avg. income 414,283 NOK for parents of first-born girls and 414,427 NOK for parents of first-born boys, $p = 0.673$.).

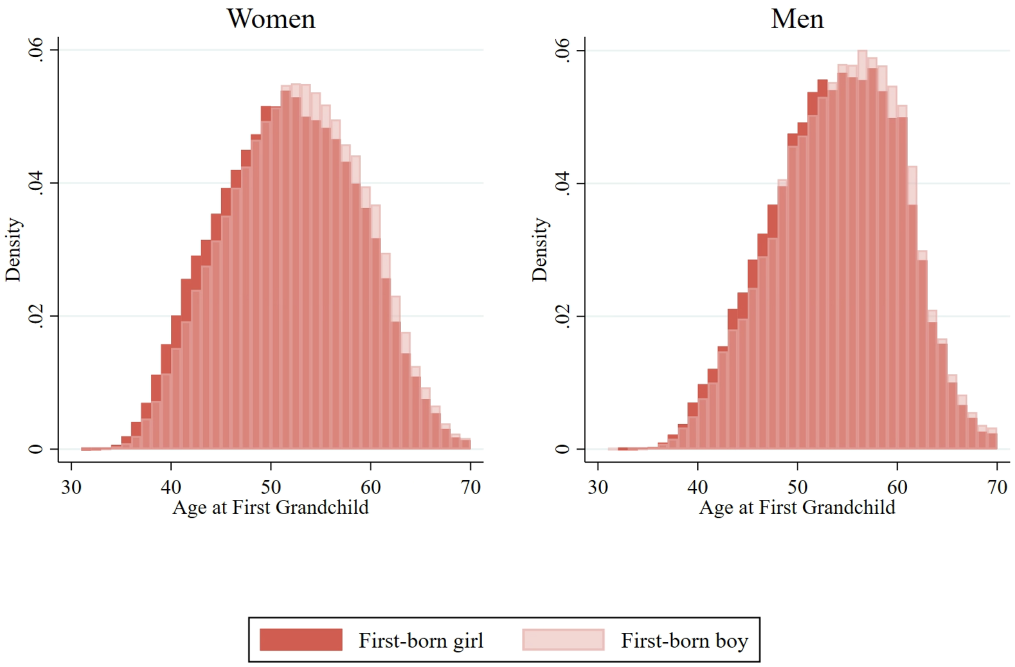

Fig. S16. Distribution of age at first grandchild for women and men, depending on the sex of their first-born child.

References

1. A Grigoryeva, Own gender, sibling's gender, parent's gender: The division of elderly parent care among adult children. *Am. Sociol. Rev.* **82**, 116–146 (2017).
2. J Compton, RA Pollak, Proximity and co-residence of adult children and their parents in the united states: Descriptions and correlates. *Annals Econ. Stat. d'Économie et de Stat.* pp. 91–114 (2015).
3. F Thomese, AC Liefbroer, Child care and child births: The role of grandparents in the netherlands. *J. Marriage Fam.* **75**, 403–421 (2013).
4. Y Zeng, MS Brasher, D Gu, JW Vaupel, Older parents benefit more in health outcome from daughters' than sons' emotional care in china. *J. aging health* **28**, 1426–1447 (2016).
5. Z Efrat, OO Akinfenwa, KH Nicolaides, First-trimester determination of fetal gender by ultrasound. *Ultrasound Obstet. Gynecol. The Off. J. Int. Soc. Ultrasound Obstet. Gynecol.* **13**, 305–307 (1999).
